# Conic curve encryption and digital signature based on complex number theory for cybersecurity applications

**DOI:** 10.1038/s41598-025-00334-6

**Published:** 2025-07-01

**Authors:** Ahmed Kamal, H. A. El-Kamchochi, Adel El-Fahar, Esam A. A. Hagras

**Affiliations:** 1 Engineering Department, Air Defense College, Alexandria University, Alexandria, Egypt; 2 Electrical Department, Faculty of Engineering, Alexandria University, Alexandria, Egypt; 3 Electronics and Communications Department, Faculty of Engineering, Delta University for Science and Technology, Gamasa, Dakahlia Egypt

**Keywords:** Elliptic curve cryptography, Conic curve cryptography, Secure image transmission, Integer factorization problem, Public key cryptosystem, Discrete logarithm problem, Pseudorandom number generator, Engineering, Mathematics and computing

## Abstract

Secure image transmission requires robust algorithms to ensure authentication, integrity, non-repudiation, and confidentiality. Addressing emerging security challenges necessitates continuous advancements in cryptographic design. This paper presents an authenticated and encrypted image scheme that achieves all essential security services. While Elliptic curve cryptography (ECC) remains a fundamental component of recent encryption schemes, it is vulnerable to side-channel and inherent ECC-specific attacks. To overcome these vulnerabilities, the proposed scheme replaces ECC with Conic curve cryptography (CCC), offering enhanced security and performance. The integration of complex number theory with CCC enables a secure complex key exchange and incorporates a robust Iterative conic curve pseudorandom number generator (ICC-PRNG) to thwart all known attack types. The system is a public key cryptosystem based on multi-hard problems, including the Gaussian conic curve integer factorization problem (GCC-IFP), Conic curve discrete logarithm problem (CC-DLP), and Conic curve integer factorization problem (CC-IFP), combined with XOR operations for image encryption. Additionally, the scheme introduces a novel complex digital signature for encrypted images, leveraging complex arithmetic to enhance security. Experimental results demonstrate high entropy 7.999, correlation near 0.0001, key space $>{2}^{688}$, and average PSNR of 8.51 dB, ensuring resilience against brute-force and statistical attacks. Additionally, the scheme achieves encryption times of 25 ms, making it suitable for real-time applications. Security analysis validates robustness against various attacks, with NIST statistical tests confirming ICC-PRNG effectiveness. By leveraging complex numbers over conic curves, the proposed method improves security and computational efficiency, establishing it as a promising solution for advanced image encryption.

## Introduction

Cybersecurity covers a wide range of practices and technologies developed to safeguard digital systems, networks, and data against unauthorized access and harmful threats.^1,2^. Protecting digital images is crucial within communication networks, particularly in sensitive applications such as healthcare, surveillance, and military communications^3,4^. Ensuring the authentication, integrity, non-repudiation, and confidentiality of transmitted images is essential to prevent misuse, data breaches, and identity theft^5^. Consequently, image encryption has emerged as a key area of research, providing solutions to safeguard images during storage and transmission^6,7^.

Image security requirements vary based on the application, with different scenarios demanding varying levels of protection. Encryption and digital signatures are essential components for long-term security in cryptographic systems^8^. Image encryption transforms the image to an unintelligible form, ensuring confidentiality^9^, while digital signatures add ownership information for authentication^10^. Achieving secure image encryption depends on integrating reliable randomness sources to ensure unpredictability. However, the quality of randomness varies among encryption systems, making the choice of a robust cryptographic method crucial^11,12^.

Traditional encryption methods, such as the Advanced Encryption Standard (AES)^13^, Data Encryption Standard (DES)^14^, and Rivest-Shamir-Adleman (RSA)^15,16^, are effective for general data but face challenges when applied to image encryption. Images exhibit unique characteristics like high redundancy and pixel correlation, which reduce the efficiency of conventional methods^17^. Moreover, modern cryptographic systems are increasingly vulnerable to emerging threats such as power analysis and timing attacks. Addressing these challenges requires innovative solutions that balance security, computational efficiency, and resilience to attacks^18–20^.

Cryptographic algorithms often derive their security from solving challenging mathematical problems like the Integer Factorization Problem (IFP) and the Discrete Logarithm Problem (DLP)^21^. RSA relies on IFP for secure digital signatures, while ECC uses DLP for its cryptographic strength^22,23^. However, advances in attack methods and resources have demonstrated that any single-problem-based cryptographic system can eventually be compromised. Designing schemes that rely on multiple hard problems ensures that even if one problem is compromised, the system remains secure^24,25^.

During the recent decade, numerous encryption schemes have been proposed to secure image transmission^26,27^. These include chaos-based systems^28,29^, elliptic curve techniques^30,31^, and hybrid approaches^32,33^. Chaos-based encryption leverages properties such as sensitivity to system parameters and initial conditions, offering strong security for image encryption. Enhancements, such as dynamic S-box generation with Henon or logistic-sine maps, quantum logistic maps for keystream generation, and additional system parameters, have improved their robustness. Non-chaos-based methods have also shown success, employing techniques like fractal-based PRNGs^34^, Feistel networks, DNA encoding for pixel transformation^35,36^, and transforms like the Discrete Wavelet Transform (DWT) and Fourier Transform for frequency-domain encryption^37,38^.

ECC has emerged as a prominent public-key cryptosystem due to its smaller key sizes, high security, and efficient computations. Despite its advantages, ECC faces challenges, particularly in resource-constrained environments. Operations on elliptic curves are computationally intensive and susceptible to side-channel attacks. Recent advancements address these limitations by incorporating Gaussian integers into ECC. This approach utilizes complex arithmetic to reduce computational overhead while enhancing security for operations like point multiplication. These innovations extend ECC’s applicability, making it more suitable for image encryption^39,40^.

CCC is an emerging paradigm offering superior computational efficiency and resistance to attacks compared to ECC. CCC leverages the properties of conic curves to implement problems like IFP, as in RSA, and DLP, as in ECC, providing robust security against various threats. Additionally, CCC introduces a novel framework that ensures computational and communication efficiency, making it highly suitable for secure systems in image encryption and digital signature schemes.

This paper introduces an authenticated RGB image encryption scheme designed for secure and efficient transmission, particularly in high-stakes applications.

### The key contributions are:

1. *Integration of complex number theory and Conic curve cryptography (CCC):* The scheme addresses vulnerabilities and inefficiencies in traditional cryptographic methods by leveraging the advanced properties of CCC and complex number theory.
2. *Innovative pseudorandom number generation:* Introduced a novel Iterative conic curve pseudorandom number generator (ICC-PRNG), which generates secure and efficient pseudorandom sequences that successfully pass all statistical randomness tests.
3. *Hybrid multi-hard problem-based digital signature:* Proposed a digital signature algorithm combining three cryptographic hard problems: Conic curve discrete logarithm problem (CC-DLP), Gaussian conic curve integer factorization problem (GCC-IFP) and conic curve integer factorization problem (CC-IFP). This ensures robust authentication, data integrity, and non-repudiation, providing long-term security.

The remainder of this paper is organized as follows: Section "Introduction" reviews the use of CCC and its counterpart schemes. Section "Preliminaries" covers the preliminaries of complex number theory and introduces the CCC. Section "Proposed scheme" details the construction of the ICC-PRNG. Section "Proposed encryption system" presents the proposed complex authenticated encryption system and complex digital signature. Section "Result analysis and system evaluation" discusses the simulation results and system evaluation. Section "Noise attack" analyzes the scheme’s resistance to noise attacks. Section "Complexity evaluation and security outcomes" presents a complexity analysis and corresponding security outcomes, supported by illustrative numerical examples. Finally, Sect.  “Conclusion” concludes the paper.

### Related work

Recent advancements in image encryption have explored elliptic curves, Gaussian integers, and emerging paradigms such as Conic curve cryptography (CCC) to improve both computational efficiency and security. Several studies have investigated enhancements to traditional cryptographic approaches, aiming to address performance bottlenecks and security vulnerabilities.

Tyagi et al.^22^ conducted a comparative study between RSA and ECC, demonstrating ECC’s computational efficiency and stronger security due to its resistance to integer factorization attacks. However, the study primarily provides theoretical analysis without practical encryption implementation or authentication mechanisms, leaving its real-world applicability unverified. Similarly, Rahnama et al.^23^ proposed replacing RSA with ECC to mitigate known vulnerabilities, emphasizing its security benefits but without addressing performance trade-offs in applied scenarios.

In the domain of image encryption, Hayat et al.^41^ introduced a two-phase encryption system using ECC for S-box construction and PRNG generation. While this approach enhances security, it does not include a built-in authentication mechanism such as a digital signature, which could improve integrity verification. Additionally, the computational overhead is not thoroughly analyzed, raising concerns about real-time feasibility. AbdElHaleem et al.^43^ designed an ECC-based PRNG framework, verified using the NIST test suite, demonstrating resilience against statistical attacks. Although the framework is validated for security, its computational efficiency is not comprehensively analyzed, and its direct impact on encryption robustness against cryptanalytic attacks remains unclear. Dawahdeh et al.^44^ proposed a hybrid encryption system combining ECC with the Hill cipher, achieving a balance between security and computational efficiency. However, the scheme inherits the Hill cipher’s susceptibility to known-plaintext attacks, which could weaken its security under specific attack scenarios.

To mitigate ECC’s computational complexity, researchers have explored Gaussian integers for cryptographic enhancements. Elsayed et al.^46^ extended ECC operations over Gaussian primes, significantly increasing security by nearly doubling the group order. However, while security is strengthened, the computational efficiency of the approach is not thoroughly assessed, raising questions about its feasibility for resource-constrained environments. Aung and Hla^47^ employed complex number arithmetic in ECC to improve robustness, yet practical implementation details and performance benchmarks remain unexplored. Safieh et al.^48^ introduced an ECC point multiplication method using Gaussian integers to resist side-channel attacks. Although effective in mitigating power analysis threats, the work does not assess its efficiency compared to standard ECC implementations, leaving its practicality uncertain. Sajjad et al.^49^ applied Gaussian integers within an SPN-based image encryption scheme, offering strong security through substitution-permutation structures. However, the scheme is purely symmetric and lacks key exchange mechanisms, limiting its applicability to scenarios requiring public-key cryptography.

Conic curve cryptography (CCC) has recently emerged as an alternative to ECC, leveraging conic curves for encryption and authentication. Abdelfatah et al.^50^ proposed an authenticated image encryption scheme integrating CCC with Mersenne Twister PRNG, achieving secure transmission through a combination of encryption and digital signatures. The scheme benefits from efficient point operations and strong security, relying on the Conic curve discrete logarithm problem (CC-DLP) and Integer Factorization Problem (IFP). However, embedding digital signatures within the cipher image may introduce additional data overhead. Daniel et al.^51^ developed a CCC-based signcryption scheme for e-payment systems, ensuring forward security and ciphertext authentication, but the work lacks performance comparisons with ECC-based counterparts. Yongnan Li^52^ proposed a tile assembly model for computing point-multiplication in conic curves over finite fields *GF*(2^*n*^), relevant to CCC arithmetic. While mathematically sound, it is limited to theoretical computation without application to real cryptographic systems or performance evaluation. Daniel et al.^53^ proposed an efficient authenticated encryption scheme based on CC-DLP and CC-IFP, ensuring forward secrecy and public verifiability using conic curve scalar multiplications. However, it lacks performance validation and comparison with ECC-based schemes, leaving its practical efficiency unverified.

These studies collectively highlight the growing role of Gaussian integers and CCC in modern cryptography. While ECC remains widely used, its computational cost has driven research toward alternative approaches that enhance efficiency without compromising security. The proposed work builds upon these developments by integrating CCC with complex number theory, offering improved security, reduced computational overhead, and enhanced suitability for resource-constrained environments^54,55^. Unlike previous ECC-based encryption approaches that focus solely on efficiency^22,23^, our method integrates both encryption and digital signature functionalities within a unified framework, enhancing both security and authentication. Furthermore, while Gaussian-integer-based cryptographic schemes^46–48^ improve security through extended group orders, they do not leverage hybrid security mechanisms as proposed in our work. Our integration of CCC and complex number theory addresses these limitations by providing a multi-hard problem security foundation, making our approach more resistant to factorization and discrete logarithm-based attacks. Table 1 provides a comparative overview of related research, highlighting the applied methods, cryptographic functionalities, underlying hard problems, and identified research gaps.

**Table 1:** Comparative overview of related schemes: applied methods, security foundations, and research gaps.

References	Encryption scheme & functionality	Utilized hard problems	Identified gaps
^22^	Comparative study of RSA & ECC, highlighting ECC’s efficiency & security advantages	EC-DLP	Theoretical analysis without practical encryption/authentication implementation
^23^	Replaces RSA with ECC for key generation, enhancing security	EC-DLP	Lacks discussion on real-world performance trade-offs
^41^	Two-phase image encryption using ECC for S-box and PRNG generation	EC-DLP	Does not include a digital signature; lacks real-time efficiency analysis
^43^	ECC-based PRNG framework validated via NIST tests	EC-DLP	Focuses on PRNG security but lacks performance analysis for encryption speed
^44^	Hybrid ECC + Hill Cipher for image encryption	EC-DLP + Hill Cipher	Inherits Hill Cipher’s vulnerability to known-plaintext attacks
^46^	ECC over Gaussian primes, increasing security	EC-DLP over Gaussian Integers	Security enhancements but lacks computational efficiency analysis
^47^	ECC using complex number arithmetic for cryptographic security	EC-DLP with Complex Numbers	No performance benchmarks or practical implementation details
^48^	ECC with Gaussian integers to resist side-channel attacks	EC-DLP + Gaussian Integers	Strong against power analysis but lacks encryption efficiency assessment
^49^	Substitution-Permutation Network (SPN) using Gaussian integers	SPN—Symmetric Cryptography	Fully symmetric system; lacks public key cryptographic mechanisms
^50^	CCC-based image encryption with Mersenne Twister PRNG & digital signature	CC-DLP + IFP	Embeds signature in cipher image but introduces additional data overhead
^51^	CCC-based signcryption for e-payment security	CC-DLP + IFP	Lacks performance comparisons with ECC
^52^	A tile assembly model for computing point-multiplication over conic curves in *GF*(2*n*)	Indirectly tied to the hardness of CC-DLP	Does not provide an actual encryption or signature scheme
^53^	Authenticated encryption with digital signature using CCC; forward secrecy, public verifiability, and ciphertext authentication	CC-DLP + CC-IFP	Lacks performance evaluation and ECC comparison; implementation feasibility unverified

## Preliminaries

In the following sections, we explore the mathematical foundations of complex number theory and its integration with CCC in details.

### Complex number theory

#### Definition 2.1

In number theory, Gaussian integers^45^ are a subset of complex numbers in which both the realcomponent and imaginary component are integers. The set of Gaussian integers is denoted by *Z*[*i*] and defined as: *Z*[*i*] = {*a* + *bi* : *a*, *b* ∈ *Z* }, where *i* = $\sqrt{-1}$.

1. **Addition and subtraction**: Gaussian integers can be added or subtracted by separately combining or subtracting their real and imaginary parts. For example, if ${Z}_{1} = a + bi$ and ${Z}_{2} = c + di$ then: ${Z}_{1} + {Z}_{2} = (a + c) + (b + d)i , {Z}_{1} - {Z}_{2} = (a - c) + (b - d)i.$
2. **Multiplication**: The multiplication of Gaussian integers follows the distributive property of complex numbers: $(a + bi) (c + di) = (ac - bd) + (ad + bc)i$.
3. **Norm**: The norm of a Gaussian integer $Z = a + bi$ is defined as the sum of the squares of its real part and imaginary part, given by: $N(Z) = {\left|Z\right|}^{2} = Z \cdot \overline{Z } = {a}^{2} + {b}^{2}$. The norm is always a non-negative integer and, $\overline{Z } = a - bi$ is the conjugate of *Z*.
4. **Reciprocal**: For a Gaussian integer $Z = a + bi$, the real part and imaginary part are employed to calculate the reciprocal as: ${Z}^{-1}$ is: ${Z}^{-1}= (a/N(Z)) - (b/N(Z))i$.
5. **Division**: To divide two complex numbers ${Z}_{1}= a + bi$ and ${Z}_{2} = c + di$, multiply the first complex number by the reciprocal of the second: ${Z}_{1}/{Z}_{2} = {Z}_{1} \cdot {Z}_{2}^{-1}$.
6. **Scalar multiplication**: To multiply a complex number $Z = a + bi$, by a scalar integer number $n$, multiply the scalar across the real part and imaginary part of the complex number: $n$ *. Z* = $n$ . *a* + $n$ . *bi*.

#### Definition 2.2

A Gaussian integer $\pi$ is called a *Gaussian prime* if for any Gaussian integers *a* and *b*, $\pi$ divides the product $a.b$, implies $\pi$ divides $a$ or $\pi$ divides $b$^46^. Symbolically,$\pi$ | *a*.*b*⟹   $\pi$ | $a$ or $\pi$ | $b$.

#### Theorem 2.1

(Classification of Gaussian Primes and the Computation of Unique Factorization)

The following elements constitute all Gaussian primes. For each type, the conditions under which a Gaussian prime divides a given Gaussian integer *a* are also specified^47^:

1. **Prime 1+ *i***: The Gaussian number $1 + i$ is recognized as a Gaussian prime. Furthermore, if $2 | N(a)$, then $1+i | a$.
2. **Real Prime **${\varvec{\pi}} \equiv$**3 mod 4**: If $\pi$ is a real prime such that $\pi$ ≡ 3 mod 4, then $\pi$ is considered as a Gaussian prime. Additionally, if $p |N(a)$, then $p | a$.
3. **Primes of the form **${\varvec{\pi}}$** = *a* + *bi***: Where $\pi$ = *a*^2^ + *b*^2^ is a prime ≡ 1 mod 4. If $\pi$ | *N*(*a*), then one or both of $\pi$ or $\overline{\uppi }$ divides *a*.
4. **Unit Multiples of the Above**: Any Gaussian integer that is a unit multiple of the above primes is also a Gaussian prime.

#### Definition 2.3

Modulo operation for gaussian integers and finite fields.

Let the set of Gaussian integers be represented by *Z*[*i*], and $Z{\left[i\right]}_{\pi }$ represents the residue class of Gaussian integers modulo π, where $\pi = a + bi$ with *a*,*b* ∈ *Z*: The modulo function $f: Z[i]\to Z{\left[i\right]}_{\pi }$ is defined for $x = c + di$ with $c,d \in Z$ as:

1 $$x\,\, mod\,\,\pi = x -\left\lfloor {\frac{\text{x}{\uppi }^{*}}{\uppi {\uppi }^{*}}} \right\rfloor \pi$$

where ${\pi }^{*}$ denotes the conjugate of π and the notation $\lfloor \cdot \rfloor$ represents rounding to the nearest Gaussian integer. To round a Gaussian integer $z \in Z[i]\pi$, the real and imaginary part’s coefficients are individually rounded to the nearest integers^48^. This ensures that each component is rounded independently to its closest integer value, maintaining the result as a Gaussian integer, thus for *x* = *c* + *di*, ⌊*x*⌋ = ⌊*c*⌋ + ⌊*d*⌋ *i*. The set of Gaussian integers defined by *G*_*p*_ = {*x* mod π: *x* = 0,…, *p* − 1, *x* ∈ *Z*} forms a finite field that is isomorphic to the prime field GF(*p*) when *p* is a prime number such that *p* ≡ 1 mod 4. Consequently, these sets are highly applicable to ECC and CCC^48^. In such cases, *p* can be represented as a sum of two perfect squares, specifically π = *a* + *bi* where *a* and *b* are integers, and *p* = ππ^∗^  = *a*^2^ + *b*^2^.

#### Theorem 2.2

(Generators of Residue Classes Modulo Gaussian Primes) Let π be a Gaussian prime^49^, and let *n*(π) represent the number of Gaussian integers modulo π. For any non-zero g ∈ *Z*[*i*], the following holds: g^*n*(π)−1^ ≡ 1 (modπ), Thus, g is a generator of the residue class modulo π.

### Conic curve cryptography

Conic curves are an emerging area of interest in cryptography due to their computational simplicity and efficiency. Unlike elliptic curves, which have been widely studied and implemented in cryptographic systems, conic curves offer a more straightforward algebraic structure that can be advantageous for certain cryptographic applications^50^. A conic curve over a finite field *F*_*p*_, in which *p* represents an odd prime number defined by the equation:

2 $$C(F_{p} ):y^{{{2} }} = ax^{{{2} }} - bx$$

where *a* and *b* are foundations of ${\mathbb{F}}_{p}^{*}$, representing the group of non-zero elements under multiplication in the finite field. The set *F*_*p*_ consists of *p* elements, typically represented as: *F*_*p*_ = {0,1,2,…,*p* − 1}, and the multiplicative group is: ${\mathbb{F}}_{p}^{*}$ = *F*_*p*_∖{0}*.* Apparently, when $x=0$, the point $O(\text{0,0})$ is the origin. For $x\ne 0$, let $t=y{x}^{-1}$ and substitute $y=xt$ in the Eq. (2). This substitution yields^51,53^:

3a $$x\left(a-{t}^{2}\right)=b,a,b\in {\mathbb{F}}_{p}^{*}$$

If $a={t}^{2}$, Eq. (3a) is not satisfied; if $a\ne {t}^{2}$, then Eq. (3a) provides:

3b $$x=b {\left(a-{t}^{2}\right)}^{-1},\,\, y=bt{\left(a-{t}^{2}\right)}^{-1}$$

where $a,b\in {\mathbb{F}}_{p}^{*}$ and ( )^−1^ represents the multiplicative inverse in ${\mathbb{F}}_{p}^{*}$. For any $t\in {\mathbb{F}}_{p}$ such that ${t}^{2}\ne a$, let $P(t)$ denote the point $(x,y)$ within $C\left({\mathbb{F}}_{p}\right)$ determined by the Eq. (3b). Additionally, an idealized point $O$, named as point at infinity $P(\infty )$ is similarly considered a point on $C\left({\mathbb{F}}_{p}\right)$.

Define:

3c $$T=\left\{t\in {\mathbb{F}}_{p};{t}^{2}\ne a\right\}\cup \{\infty \}$$

then, $P:T\to C\left({\mathbb{F}}_{p}\right)$ is a bijective mapping. Following^52,56^, let us outline the addition $\oplus$ of points in $C\left({\mathbb{F}}_{p}\right)$. For all points $\forall P(t)\in C\left({\mathbb{F}}_{p}\right)$ and $t\in T$, so that

3d $$P(t)\oplus P(\infty )=P(\infty )\oplus P(t)$$

Assume $P\left({t}_{1}\right),P\left({t}_{2}\right)\in C\left({\mathbb{F}}_{p}\right)$, where ${t}_{1},{t}_{2}\in T$ and ${t}_{1},{t}_{2}\ne \infty$,such that

3e $$P\left({t}_{1}\right)\oplus P\left({t}_{2}\right)=P\left({t}_{3}\right)$$

where

3f $${t}_{3}=\left\{\begin{array}{l}\left({t}_{1}{t}_{2}+a\right){\left({t}_{1}+{t}_{2}\right)}^{-1},{t}_{1}+{t}_{2}\ne 0,\\ \infty ,{t}_{1}+{t}_{2}=0.\end{array}\right.$$

It is evident that, ${t}_{3}\in T$, and the operation $\oplus$ is commutative^50^. For any point $P(t)\in C\left({\mathbb{F}}_{p}\right)$, the negative element is given by: $-P\left(\infty \right)=P\left(\infty \right), -P(t)=P(-t)$, From Eqs. (3c) to (3e), it can be readily shown that for all $\forall P\left({t}_{1}\right),P\left({t}_{2}\right),P\left({t}_{3}\right)\in C\left({\mathbb{F}}_{p}\right)$,

3g $$\left(P\left({t}_{1}\right)\oplus P\left({t}_{2}\right)\right)\oplus P\left({t}_{3}\right)=P\left({t}_{1}\right)\oplus \left(P\left({t}_{2}\right)\oplus P\left({t}_{3}\right)\right)$$

A noteworthy feature of CCC is the simplicity of both the encryption and decryption processes^52^. Denote $\text{T}\setminus \{\infty \}$ as ${\text{T}}^{*}$, and assume a message $t\in {\text{T}}^{*}$, Here is how to perform the encryption and decryption for this message using CC .

Encryption:

4a $$\begin{array}{c}P(t)=\left({X}_{t},{Y}_{t}\right)\\ \left\{\begin{array}{l}{X}_{t}=b{\left(a-{t}^{2}\right)}^{-1}\,\, mod\,\,n\\ {Y}_{t}=bt{\left(a-\text{t}\right)}^{-1}\,\, mod\,\, n\end{array}\right.\end{array}$$

Decryption:

4b $$t={Y}_{t}\cdot {X}_{t}^{-1}\,\, mod \,\,p$$

### The role of conic curves in efficient and secure cryptographic systems

The primary advantage of conic curves in cryptographic systems lies in the simplicity of their group operations. Point addition and scalar multiplication on conic curves are computationally less intensive than those on elliptic curves. This makes conic curves particularly suitable for devices with limited computational resources^50,51,69^. Conic curves can be integrated into various cryptographic protocols that traditionally use elliptic curves. Examples include:

1. **Public key cryptosystems**: Conic curves can be utilized to construct public key algorithms analogous to RSA and El-Gamal. The reduced complexity of point operations can result in more rapid encryption and decryption processes^56,57^.
2. **Key exchange protocols**: In a manner analogous to the Diffie-Hellman key exchange protocol, conic curves can facilitate secure key exchange with lower computational overhead^58^.
3. **Digital signatures**: The properties of conic curves allow for the implementation of digital signature schemes that are both efficient and secure^59,60^.

### Security of conic curves over rings

The conic curve *C*_*n*_(*a*,*b*) defined by the equation: ${y}^{2} \equiv a{x}^{2} - bx (mod n)$ where $a, b \in {Z}_{n}$, and $n = pq$ with $p$ and $q$ being large odd primes. Here, $n, a$, and $b$ are coprime, and $p+1 = 2r$ and $q+1=2s$ for some integers $r$ and $s$. Additionally,$a (mod p) \equiv a (mod q) \equiv -1$. The order of the curve ${C}_{n}(a,b)$, denoted as *N*_*n*_, is given by the least common multiple of the orders of the finite fields ${F}_{p}$ and ${F}_{q}: {N}_{n} = lcm\{|{C}_{p}(a,b) |,|{C}_{q}(a,b) |\}=2rs$. If $G$ is a base point on ${C}_{n}(a,b)$, a subgroup $S$ of ${C}_{n}(a,b)$ is defined as: $S = \{0,G,2G,\dots ,\left({N}_{n}-1\right)G\}$ This subgroup is produced by the base point $G$^61–63^.

#### Conic curve discrete logarithm problem (CC-DLP)

The CC-DLP is characterized as follows: Given $G$ and $kG(mod n)$, finding the value of $k$ is computationally infeasible. The security of the CC-DLP is well-supported in the literature^52,53,64^. This problem underpins the cryptographic strength of systems utilizing conic curves, analogous to the Elliptic Curve Discrete Logarithm Problem (ECDLP) in ECC^65,66^.

#### Conic curve integer factorization problem (CC-IFP)

The strength of the RSA algorithm is fundamentally dependent on the challenge of solving the IFP, which involves finding the order of the multiplicative group,$\varphi (n) = (p-1)(q-1)$. This is computationally equivalent to factorizing *n* into its prime components $p$ and $q$^45,67,68^.

A similar security foundation applies to conic curves. It has been proven^50^ that determining the order ${N}_{n}=lcm(p+1,q+1)$ of the conic curve ${C}_{n}(a,b)$ is computationally equivalent to factorizing $n=pq$. This equivalence ensures that breaking the conic curve cryptographic system would require solving the IFP, a problem considered hard in computational number theory. Furthermore, it has been shown in^51^ that ${C}_{n}(a,b)$ always possesses points *G* whose order is:${N}_{n} = lcm(|{C}_{p}(a,b) |, |{C}_{q}(a,b)|) = 2rs$.

This is a crucial requirement for digital signature algorithms relying on ${C}_{n}(a,b)$, making them more viable. In contrast, achieving this condition for general elliptic curves $E{C}_{n}(a,b)$ is not straightforward, limiting their applicability. The broader selection criteria and the simpler, faster calculations associated with conic curves ${C}_{n}(a,b)$ provide substantial advantages over elliptic curves $E{C}_{n}(a,b)$. Despite these computational benefits CCC offers security equivalent to ECC for the same key lengths. A comprehensive and detailed analysis of CCC related work is provided in^54,61,69^.

### Framework of EC-CC based PRNGs

In any encryption system, a Pseudorandom Number Generator (PRNG) is essential as it generates the pseudorandom keystream necessary for encryption. A well-designed PRNG must exhibit high sensitivity to its initial conditions, produce uniformly distributed output bits, and have a sufficiently long period to prevent cryptographic attacks. In recent schemes, researchers have utilized EC as the main source of PRNGs. There are two main types of EC based PRNG designs: Iterative Elliptic Curve Pseudorandom Number Generator (IEC-PRNG) and non-iterative designs. In the iterative design, points are generated individually, while in the non-iterative design, the points are all generated simultaneously^41^. The proposed scheme adopts the iterative design based on CC operations, which enhances the algorithm’s speed and security due to the simplicity and robustness of CC properties^50^.

Table 2 provides a comparative analysis of IEC-PRNG designs and the proposed ICC-PRNG design. The key steps for generating random sequences in the proposed system and other recent systems are as follows:

1. **Parameter selection:** In this step, the main parameters of the chosen curve are selected to derive the initial secret point and the Generator (G) point between the two parties. These initial points serve as the secret keys exchanged between the parties.
2. **Points generation:** During this step in iterative designs, points are generated individually for each iteration using the properties of the curve equation. This process generally relies on group operations on the curve, specifically point doubling and addition, with complex generators requiring more extensive curve operations.
3. **Points management:** The generated points are managed according to the design criteria. For instance, their coordinates may be converted to binary form or used to produce various numbers through mathematical transformations.
4. **Random bit extraction:** This step manipulates the output of the preceding stage to produce the desired pseudo-random bits. It involves a series of operations, such as bit truncation, padding, and manipulation, to meet the design specifications.

**Table 2:** A comparative analysis of ICC and IEC designs.

	ICC design	IEC design	Pros of CC
Curve selection	Randomly selected CCs	Secure known ECs/randomly selected ECs	Wider range of CC
Prime numbers	Large order primes $p$, $q$ to get $n$	Large order prime $p$	Use Gaussian primes
Generated points	Cyclic subgroup based CC point operations	Point operation over EC cyclic subgroup	Point add is easy and fast
Period	Hopping around the starting points $P\left({t}_{1}\right), P({t}_{2})$	Generally around the order of the G point	Provide point hopping
Suggested applications	Predetermined or arbitrary data length (e.g., video streaming, images, etc.)	Known/ Unknown data length (e.g., video streaming, images, etc.)	Suitable for limited resources

Table 3 outlines a framework of various system methodologies that implement the described steps with specific variations, when compared to the proposed method detailed in Table 6.

**Table 3:** Recent IEC-PRNG methods framework.

Ref. No	Ref.^13^	Ref.^31^	Ref.^43^
Parameters selection	Take a point ${P}_{0}$ with order $n$ Let $r\in [1,n-1]$ Let ${\alpha }_{1},\dots ,{\alpha }_{p}$ serves as the base of ${F}_{{2}^{p}}$	Selection of secure EC Selection of a point P ${S}_{0}=X(P)$	Selection of secure EC Selection of the key K ${P}_{0} = KG$
Points generation	${P}_{k}={r}^{k}{P}_{0}$ ${x}_{k}=X\left({P}_{k}\right)$	$\varphi$: truncation function $H$: hash function ${S}_{i}=\varphi \left(x\left[{S}_{i-1}P\right]\right)$ ${h}_{i}=\varphi \left(H\left({S}_{i}\right)\right)$	Increment index $n$ ${P}_{n+1} = {P}_{n} + {P}_{0}$
Points management	Writing ${x}_{k}$ $={s}_{k}^{(1)}{\alpha }_{1}+\cdots +{s}_{k}^{(p)}{\alpha }_{p}$	Utilize $\varphi$ on the $x$-coordinate of ${S}_{i-1}P$ Apply $\varphi$ on $H\left({S}_{i}\right)$	Transform the $x$, $y$ coordinates of the point ${P}_{n}$ into binary format
Random bit extraction	Read the sequence ${s}_{k}^{(i)}, i=1,\dots ,p$	Obtain the least significant bits from ${h}_{i}$	Extract the lower order 96 bits from $x,$ $y$ coordinates Combine the bits from both sets
Notes	$n$ is characterized by a large prime order $r$ holds a large multiplicative order $mod n$	The hash function strengthens the statistical characteristics of the generated bits	Any secure curve can be utilized K has a minimum length of 128 bits

## Proposed scheme

The CC cryptosystem is introduced as a versatile public key cryptosystem. Unlike elliptic curve-based cryptography, it does not require specific conic curves, making it suitable for a broad range of applications. This approach leverages the computational simplicity of CC over ECs, resulting in significant improvements in both efficiency and processing speeds. Furthermore, as explained in Sect. "Conic curve cryptography", the CC cryptosystem offers stronger protection compared to its counterparts.

### Gaussian conic curve (GCC) public key

The proposed scheme integrates a double-layer authentication mechanism to enhance security. The first layer encrypts the shared key using CC encryption, while the second layer employs Gaussian GCC encryption based on complex number theory to secure key exchange between communicating parties. Both rational CC encryption and GCC encryption utilize the same public parameters for key sharing, ensuring that no redundant data is introduced into the network. This approach mitigates side-channel attacks and ensures long-term security, making it particularly suitable for applications requiring highly secure key sharing. Table 4 outlines the Gaussian key-sharing protocol between the sender and receiver, utilizing Gaussian primes for secure key exchange. For clarity, Table 5 provides a comprehensive list of all symbols and abbreviations referenced throughout this section. The detailed steps for applying GCC-based encryption and decryption are described below:

**Table 4:** Gaussian key sharing.

Sender (User A)	Shared data	Receiver (User B)
KEY Mapping ${t}_{1} \to T \to P\left({t}_{1}\right)= \left({X}_{t1}, {Y}_{t1}\right)$ ${t}_{2}\to G\to P({t}_{2}) = ({X}_{t2}, {Y}_{t2})$		Receives data $\boxed \to$ Receive *T''* and *G''*
Point conversion ${T}^{{{\prime}}}= \left[{X}_{t1} \left(1\right)+ {X}_{t1}\left(2\right)i, {Y}_{t1}\left(1\right)+ {Y}_{t1}\left(2\right)i\right]$ $G{^{\prime}} = [{X}_{t2}(1) + {X}_{t2}(2)i, {Y}_{t2}(1) + {Y}_{t2}(2)i]$	$n$ $e$ $a$	Gaussian decryption T' = D(*T''* ) = (*d* ⋅ *T''* ) *mod n* G' = D(*G''* ) = (*d* ⋅ *G''* ) *mod n*
Gaussian encryption Sends $T{^{\prime}}{^{\prime}} =\text{E}(T{^{\prime}}, e) mod n$ Sends $G{^{\prime}}{^{\prime}} =\text{ E}(G{^{\prime}}, e) mod n$ $\boxed \to$	${T}^{{{\prime}}{{\prime}}}{ \to }$ $G{^{\prime}}{^{\prime}}{ \to }$	Key reconstruction $T\to [(\left|{X}_{{t}_{1}}\left(1\right)\right|)\| (\left|{X}_{{t}_{1}}\left(2\right)\right|), {(|Y}_{{t}_{1}}(2)|)\| ({|Y}_{{t}_{1}}(2)|)]$ $G\to [(\left|{X}_{{t}_{2}}\left(1\right)\right|)\| (\left|{X}_{{t}_{2}}\left(2\right)\right|), {(|Y}_{{t}_{2}}(2)|)\| ({|Y}_{{t}_{2}}(2)|)]$ $T \to \left({X}_{t1}, {Y}_{t1}\right)\to P\left({t}_{1}\right)\to {t}_{1}$ $G\to \left({X}_{t2}, {Y}_{t2}\right)\to P\left({t}_{2}\right)\to {t}_{2}$

**Table 5:** List of abbreviations for Sect. "Proposed scheme".

Symbol	Definition
$p,q$	Large, distinct Gaussian prime numbers
$n$	complex modulus formed by two Gaussian primes $p$ and $q$
$a,b$	Conic curve coefficients defining the shape of the curve
${C}_{N(n)}(a,b)$	Conic curve equation ${y}^{2}=a{x}^{2}-bx mod N(n)$
$N(n)$	Norms of the complex modulus $n$ used in encryption
$N(p),N(q)$	Norms of the Gaussian primes $p$ and $q$
$\#{C}_{N\left(p\right) }(a,b), \#{C}_{N\left(q\right)}(a,b)$	Cardinality (number of points) of the conic curve over $N(p)$ and $N(q)$
${N}_{N(n)}$	Least common multiple (LCM) of the conic curve cardinalities
$G$	Base point on the conic curve used in cryptographic operations
$e,d$	Public and private key pair for encryption and decryption
$kG$	Scalar multiplication of the base point $G$ in CC-DLP
$T=P\left({t}_{1}\right),G=P\left({t}_{2}\right)$	Conic curve points used for key exchange
${t}_{1}, {t}_{2}$	Exchanged private initial conditions for ICC
${T}^{{{\prime}}},{G}^{{{\prime}}}$	Complex representation of conic curve points
${T}^{{{\prime}}{{\prime}}},{G}^{{{\prime}}{{\prime}}}$	Encrypted versions of ${T}^{{{\prime}}},{G}^{{{\prime}}}$ using complex number theory
$E\left({T}^{{{\prime}}}\right)=e. {T}^{{{\prime}}}$	Encryption function encrypt ${T}^{{{\prime}}}$ using the Gaussian *mod* function
$D\left({T}^{{{\prime}}{{\prime}}}\right)=d\cdot {T}^{{{\prime}}}$	Decryption function retrieving ${T}^{\prime}$ using the Gaussian mod function

#### Private key generation and sharing

1. Conic curve selection: Choose a conic curve equation ${C}_{N(n)}:{y}^{2}=a{x}^{2}+bx \,\,mod\,\, N(n)$, where $a,b$ are curve parameters and Gaussian primes $p={x}_{1}+{y}_{1}i,q={x}_{2}+{y}_{2}i$ are selected.
2. Compute the norm: Calculate $n=p\cdot q$, ensuring that $(a,N(n))=(b,N(n))=1$, where $N(n)$ represents the norm of $n$.
3. Verify curve conditions: Ensure that $a/N(p)=a/N(q)=-1$, where $N(p)$ and $N(q)$ denote the norms of the Gaussian primes $p$ and $q$.
4. Calculate the curve order: Compute ${N}_{N(n)}=lcm\left(\#{C}_{N(p)}(a,b),\#{C}_{N(q)}(a,b)\right)$, where $\#{C}_{N(p)}(a,b)$ and $\#{C}_{N(q)}(a,b)$ denote the cardinalities of the respective conic curves.
5. Public and private key generation:
  Select an integer $e$ that is coprime to ${N}_{N(n)}$.
  Compute the private key $d$ such that $e\cdot d\equiv 1\,\, mod \,\,{N}_{N(n)}$.

Parameter distribution: Publicly share the GCC domain parameters $(a,e,n)$ while keeping $\left(d,p,q,\#{C}_{N(p)}(a,b),\#{C}_{N(q)}(a,b)\right)$ private.

#### Encryption process (Sender A)

1. Key encryption:
  Generate a shared secret key ${t}_{1}$ and encrypt it using Eq. 4b to obtain the CC point $T=$ $\left({X}_{{t}_{1}},{Y}_{{t}_{1}}\right)$, which lies on the conic curve ${C}_{N(n)}(a,b)$.
2. Convert to complex form:
  Extract real and imaginary components of $T$ :
    $${T}^{{{\prime}}}=\left({X}_{{t}_{1}}(1)+{X}_{{t}_{1}}(2)i,{Y}_{{t}_{1}}(1)+{Y}_{{t}_{1}}(2)i\right)$$
3. Generate a second shared key:
  Compute the SHA-128 hash of the plaintext RGB image to derive a second shared key ${t}_{2}$.
  Repeat steps 1 and 2 to obtain the CC point $G$ and its complex form ${G}^{\prime}$.
4. Apply gaussian encryption:
  Encrypt ${T}^{\prime}$ using the receiver’s public key $e$ and Gaussian $mod\,\, n$ using Eq. 1:
    $${T}^{{\prime}{\prime}}=E\left({T}^{\prime}\right)=e\cdot {T}^{\prime}$$
  Transmit the encrypted complex point ${T}^{{\prime}{\prime}}$ to receiver B.
5. Encrypt second key:
  Similarly, encrypt ${G}^{\prime}$ to obtain the complex ciphertext ${G}^{{\prime}{\prime}}$.

#### Decryption process (Receiver B)

1. Decrypt complex point:
  Retrieve ${T}^{\prime}$ from the received ciphertext ${T}^{{\prime}{\prime}}$ using the private key $d$ :
    $${T}^{{{\prime}}}=D\left({T}^{{{\prime}}{{\prime}}}\right)=d\cdot {T}^{{{\prime}}{{\prime}}}$$
  Since $d\cdot {T}^{{\prime}{\prime}}=d\cdot e\cdot {T}^{\prime}$, the encryption is reversed.
2. Extract real and imaginary parts:
  Compute the modulus of each component:
    $$\left|{X}_{{t}_{1}}(1)\right|,\left|{X}_{{t}_{1}}(2)\right|,\left|{Y}_{{t}_{1}}(1)\right|,\left|{Y}_{{t}_{1}}(2)\right|$$
3. Reconstruct the original CC point:
  Concatenate the real and imaginary parts to retrieve:
    $${X}_{{t}_{1}}=\left|{X}_{{t}_{1}}(1)\right|\Vert \left|{X}_{{t}_{1}}(2)\right|, {Y}_{{t}_{1}}=\left|{Y}_{{t}_{1}}(1)\right|\Vert \left|{Y}_{{t}_{1}}(2)\right|$$
  Form the original CC point:
    $$T=\left({X}_{{t}_{1}},{Y}_{{t}_{1}}\right)$$
4. Recover the shared key:
  Decrypt $T=\left({X}_{{t}_{1}},{Y}_{{t}_{1}}\right)$ using Eq. (4a) to obtain the original secret number ${t}_{1}$.
5. Decrypt second key:
  Repeat steps 1–4 for ${G}^{\prime}$ to retrieve ${t}_{2}.$

### Proposed ICC‑PRNG

The primary source for the proposed ICC-PRNG is the CC points. Initially, the sender and receiver exchange the initial secret keys to share generator points of large order, after which, CC group operations are applied to compute new points. The coordinates of these points are then converted into random bits. In the ICC-PRNG scheme, the first step involves secret key sharing (${t}_{1}$ and ${t}_{2}$) between the two parties using the CC and GCC properties as explained in Sect. "Gaussian conic curve (GCC) public key". The CC points are generated using ${t}_{1}$ and ${t}_{2}$ through CC point addition using Eq. 4a until the desired number of points is reached, where each ${t}_{n}$ number represents a point lies on the CC ($n=1, 2, 3, \dots .)$. This construction of a cyclic subgroup of points forms the proposed ICC-PRNG. Changing the initial points alters all subsequent generated points.

The coordinates ($x$, $y$) of each generated CC point are converted into a 256-bit binary format. Specifically, the lower 128 bits (LSBs) of the $x$-coordinate are padded into the 128 LSBs of $y$, while the most significant bits (MSBs) of both are discarded to avoid similarities in the generated sequence. Each point produces 256 random bits, sufficient to encrypt approximately 32 grayscale pixels $(256/8=32)$ or 10 color pixels $(256/24\approx 10)$. The proposed ICC-PRNG improves upon recent IEC schemes by offering both faster processing and enhanced security through the integration of CC and GCC. The simplicity of CC point addition significantly accelerates computations, while the use of Gaussian number theory strengthens resistance against various attacks. Decimal coordinates are converted to binary during point management, and binary bit manipulation ensures high randomness in the extracted bits. Consequently, the ICC-PRNG generates a highly Random binary sequence (RBS), making it well-suited for secure image encryption.

In practical implementation of the ICC-PRNG, the choice of the starting points $P({t}_{1})$ and $P({t}_{2})$ are selected randomly for each round and are of high order. The prime numbers used must be complex and large enough to be suitable for cryptographic applications. In the proposed scheme, a 256-bit CC is used; the choice of the CC and its order depends on the application. The proposed scheme demonstrates enhanced security and efficacy, making it well-suited for resources-conistrained applications.

## Proposed encryption system

The proposed encryption system integrates both encryption and Complex Digital Signatures (Complex-DS) to ensure long-term security and robustness against cryptographic attacks. A secure encryption algorithm should adhere to Shannon’s principles of confusion and diffusion, which are achieved in the proposed system through two primary phases:

1. RGB image encryption and complex-DS generation
2. Complex-DS verification and RGB image decryption

**Fig. 1:**
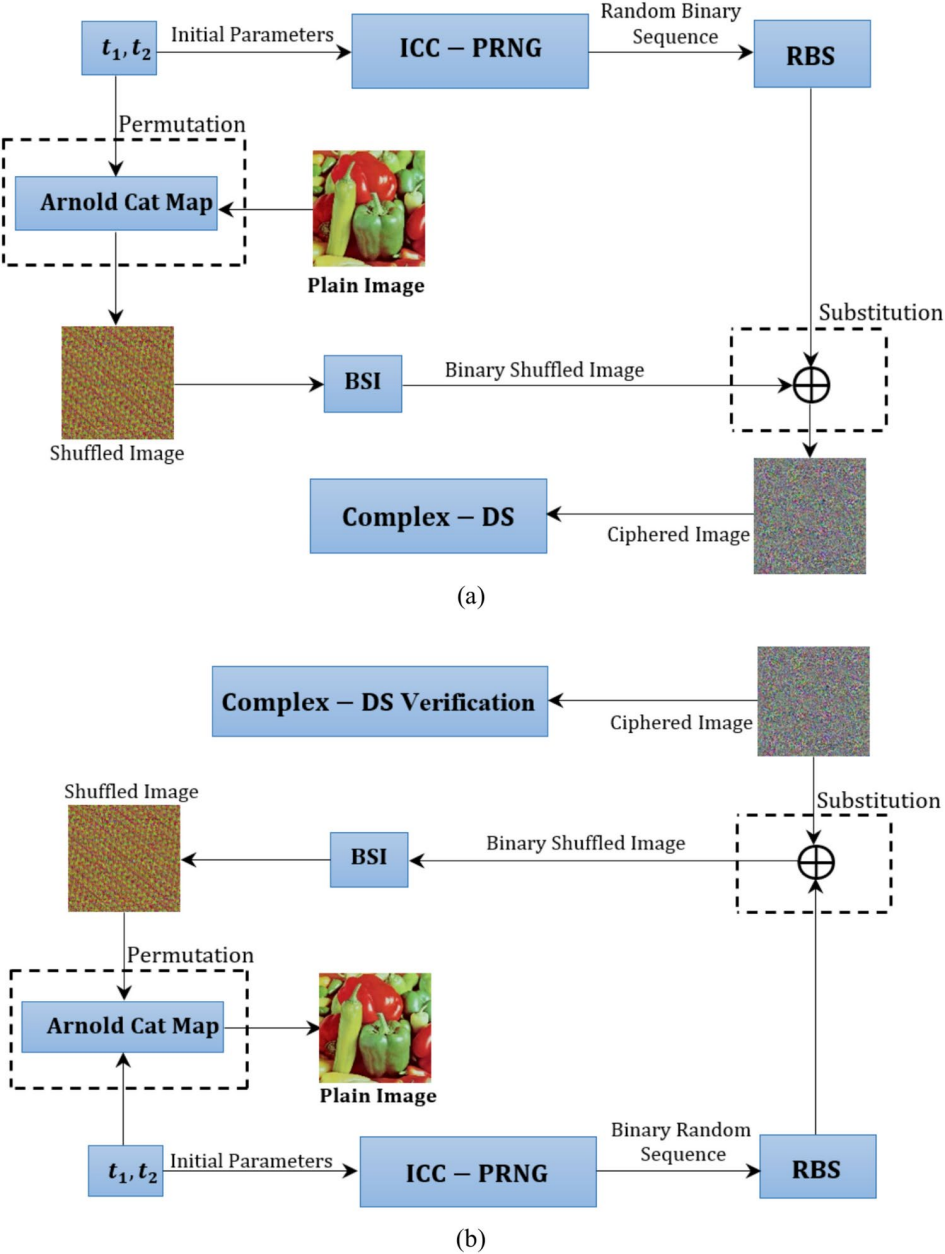
Block diagram for the proposed scheme (**a**) encryption (**b**) decryption.

In the encryption phase, permutation is applied to pixel positions using the Arnold Cat Map (ACM), followed by substitution via Iterative Conic Curve-based Pseudorandom Number Generator (ICC-PRNG). Additionally, a Complex-DS is generated, leveraging complex number theory and conic curve (CC) parameters to ensure authentication and integrity. A hash function is applied to the encrypted image for additional security. The system is sensitive to input alterations. Figure 1 provides an illustration of the block diagram for the proposed scheme.

### Encryption and complex digital signature generation

Given an RGB image of dimensions M × N, the encryption process is structured as follows:

**Step 1:** Image Layer Separation

- The image is decomposed into its three-color channels: Red (R), Green (G), and Blue (B).

**Step 2:** Permutation using Arnold Cat Map (ACM)

- The pixel locations of each channel are permuted using ACM, defined by:
  5 $$\left[\begin{array}{l}{x}^{{{\prime}}}\\ {y}^{{{\prime}}}\end{array}\right]=\left[\begin{array}{cc}1& a\\ b& (ab+1)\end{array}\right]\left[\begin{array}{l}x\\ y\end{array}\right] mod\,\, M$$
  where:
  - $\left({x}^{\prime},{y}^{\prime}\right)$ are the permuted pixel positions.
  - $(x,y)$ are the original positions.
  - $M$ is the image matrix size.
  - $a,b$ are derived from ICC-PRNG as:
    6a $$a = \left(\left(H\left[{t}_{1}\right]+{k}_{l}\right) \,\,mod\,\, M-1\right)+1$$
  6b $$b = \left(\left(H\left[{t}_{2}\right]+{k}_{l+3}\right)\,\, mod \,\,M-1\right)+1$$
  where
  - $H\left[{t}_{1}\right]$ and $H\left[{t}_{2}\right]$ are hash values of the shared secret keys ${t}_{1}$ and ${t}_{2}$.
  - The value ${k}_{l}$ represents the chosen division of the first output sequence from the ICC-PRNG.

**Step 3:** Substitution using ICC-PRNG output sequence.

- Each pixel undergoes bitwise XOR encryption:
  7a $$E[R] = {P}_{R}[j] \oplus RS[j]$$
  7b $$E[G] = {P}_{G}[j] \oplus RS[j+1]$$
  7c $$E[B] = {P}_{B}[j] \oplus RS[j+2]$$
  where:
  - $E[R],E[G],E[B]$ are the encrypted pixel values.
  - ${P}_{R}[j],{P}_{G}[j],{P}_{B}[j]$ are the original pixel values.
  - $RS[j]$ is the random sequence bitstream from ICC-PRNG.

**Step 4:** Formation of the Ciphered Image.

- The encrypted R, G, and B layers are recombined into the final ciphered image $C$.

**Step 5:** Public Key Generation for Digital Signature.

- The sender computes the public key ${U}_{A}:$
  8 $${U}_{A}={Z}^{V} mod N(n)\cdot G=\left({x}_{A},{y}_{A}\right)$$
  where:
  $Z$ is chosen such that $Z \in \{1,. .., n - 1\}$ and $\text{gcd}(Z, n)=1$*,* $G$ is a securely exchanged point.
- $V$ is the sender’s private key.
- Convert ${U}_{A}$ into complex form:
  9 $${U}_{A}^{{{\prime}}}=\left({x}_{A}\left(1\right)+{x}_{A}\left(2\right)i,{y}_{A}\left(1\right)+{y}_{A}\left(1\right)i\right)$$
  where the values ${x}_{A}$ and ${y}_{A}$ each consists of 128 bits. The first 64 bits from ${x}_{A}$ and ${y}_{A}$ are denoted as ${x}_{\text{A}}\left(1\right),{y}_{A}(1)$, respectively, while the remaining 64 bits are denoted as ${x}_{A}\left(2\right),{y}_{A}(2)$, respectively.

**Step 6:** Complex Digital Signature Generation

- The sender splits $V$ into two random integers: $\alpha$ and $\beta$, such that:
  $$V=\alpha +\beta$$
- The digital signature components are computed as follows:
  9a $$R={Z}^{\alpha } \,\,mod\,\, N\left(n\right)\cdot G=\left({x}_{R},{y}_{R}\right)$$
  9b $$\begin{array}{c}\\ S=\left(\alpha +\text{Hash}\left(C\right)+N\left(n\right)\right) mod \,\,{N}_{N(n)}\end{array}$$
- Convert $R$ into complex form:
  10 $$R{^{\prime}}=\left({x}_{R}(1)+{x}_{R}(2)i,{x}_{R}(1)+{y}_{R}(2)i\right)$$
  where the values ${x}_{R}$ and ${y}_{R}$ each consists of 128 bits. The first 64 bits from ${x}_{R}$ and ${y}_{R}$ are denoted as ${x}_{\text{R}}\left(1\right),{y}_{R}(1)$, respectively, while the remaining 64 bits are denoted as ${x}_{R}\left(2\right),{y}_{R}(2)$, respectively.
- The receiver’s public key $(e,n)$ is used to encrypt:
  $${R}^{{{\prime}}{{\prime}}}=e\cdot {R}^{{{\prime}}}$$
  $${U}_{A}^{{{\prime}}{{\prime}}}=e\cdot {U}_{A}^{{{\prime}}}$$
  This process ensures that the multi-hard problem-based digital signature and the public key ${( R}^{{\prime}{\prime}},{U}_{A}^{{\prime}{\prime}})$ are are securely transmitted in complex form.
  Notably, the proposed scheme employs the same set of parameters $(n,p,q)$ for both complex encryption and conic curve operations, thereby streamlining implementation and avoiding additional parameter overhead.
- The sender transmits ${(C, R}^{{\prime}{\prime}},S,{U}_{A}^{{\prime}{\prime}})$

### Complex digital signature verification and image decryption

Upon receiving the encrypted image $C$ and digital signature $(R^{{\prime}{\prime}},S)$ the receiver performs the following steps:

**Step 1:** Signature verification

- The receiver’s decrypt $(R^{{\prime \prime }} ,U_A^{{\prime \prime}} )$ using decryption key $d$:
  $$R=d\cdot {R}^{{{\prime}}{{\prime}}}$$
  $${U}_{A}=d\cdot { U}_{A}^{{\prime}{\prime}}$$
  Compute:
  11a $${D}_{1}={Z}^{S}\,\, mod\,\,N\left(n\right)\cdot R$$
  11b $$\begin{array}{c}\\ {D}_{2}={Z}^{\text{Hash }(E)+N(n)}\,\,mod\,\,N(n)\cdot {U}_{A}\end{array}$$
- If ${D}_{1}={D}_{2}$, authentication is successful, and decryption proceeds. Otherwise, the process is aborted.

**Step 2:** Extract RGB image layers

- The encrypted image $C$ is decomposed into its $\text{R},\text{G}$, and B components.

**Step 3:** Inverse substitution using ICC-PRNG

- The original pixel values are recovered via XOR:
  12a $${P}_{R}\left[j\right]=E\left[R\right]\oplus RS\left[j\right]$$
  12b $${P}_{G}[j]=E[G]\oplus RS[j+1]$$
  12c $${P}_{B}[j]=E[B]\oplus RS[j+2]$$

**Step 4:** De-permutation using inverse Arnold Cat Map

- The original pixel locations are restored using:
  13 $$\left[\begin{array}{l}x\\ y\end{array}\right]=\left[\begin{array}{cc}1& -a\\ -b& (ab+1)\end{array}\right]\left[\begin{array}{l}{x}^{{{\prime}}}\\ {y}^{{{\prime}}}\end{array}\right] mod\,\, M$$
- The parameters $a,b$ are retrieved using the same ICC-PRNG sequence.

**Step 5:** Image reconstruction

- The R, G, and B layers are merged to reconstruct original image.

The proposed system offers long-term security based on multi-hard problems, ensuring robustness against various attacks while being suitable for real-time applications. It fulfills all necessary security services, making it applicable across a range of fields.

### Verification of complex-DS

$$\begin{aligned}&{D}_{1}={Z}^{S} \cdot R \,\,mod \,\,N(n) \cdot G\\&\quad\,\,={Z}^{S} \cdot {Z}^{\beta }\,\, mod\,\, N(n) \cdot G \\&\quad\,\,={Z}^{\left(\alpha +\text{Hash}\left(C\right)+N(n)\right)} \cdot {Z}^{\beta }\,\, mod \,\,N(n) \cdot G \\&\quad\,\,={Z}^{\left(\alpha +\beta +\text{Hash}\left(C\right)+N(n)\right)}\,\, mod\,\, N(n) \cdot G\\&\quad\,\, ={Z}^{\left(\text{V}+\text{Hash}\left(C\right)+N(n)\right)}\,\, mod \,\,N(n) \cdot G \\&{D}_{2}={Z}^{\text{Hash}\left(C\right)+N(n)}\,\, mod \,\,N\left(n\right) \cdot {U}_{A} \\ &\quad\,\,={Z}^{\text{Hash}\left(C\right)+N\left(n\right)} \cdot {Z}^{V}\,\, mod\,\, N(n) \cdot G \\&\quad\,\,={Z}^{\left(\text{V}+\text{Hash}\left(C\right)+N(n)\right)} \,\,mod\,\, N(n) \cdot G\end{aligned}$$

## Result analysis and system evaluation

This section initially presents a thorough assessment of the randomness and efficiency of the ICC-PRNG. Subsequently, the encryption system is assessed for its performance and security. The following specifications describe the laptop on which the simulations were conducted: Intel(R) Core(TM) i7-4910MQ CPU clocked at 2.90 GHz, 16 GB RAM, operating on a 64-bit Windows 10 system, utilizing MATLAB R2022b. Small parameters were selected for the simulation to ensure manageability and efficiency. The sample images used for the simulation were obtained from^56^.

**Fig. 2:**
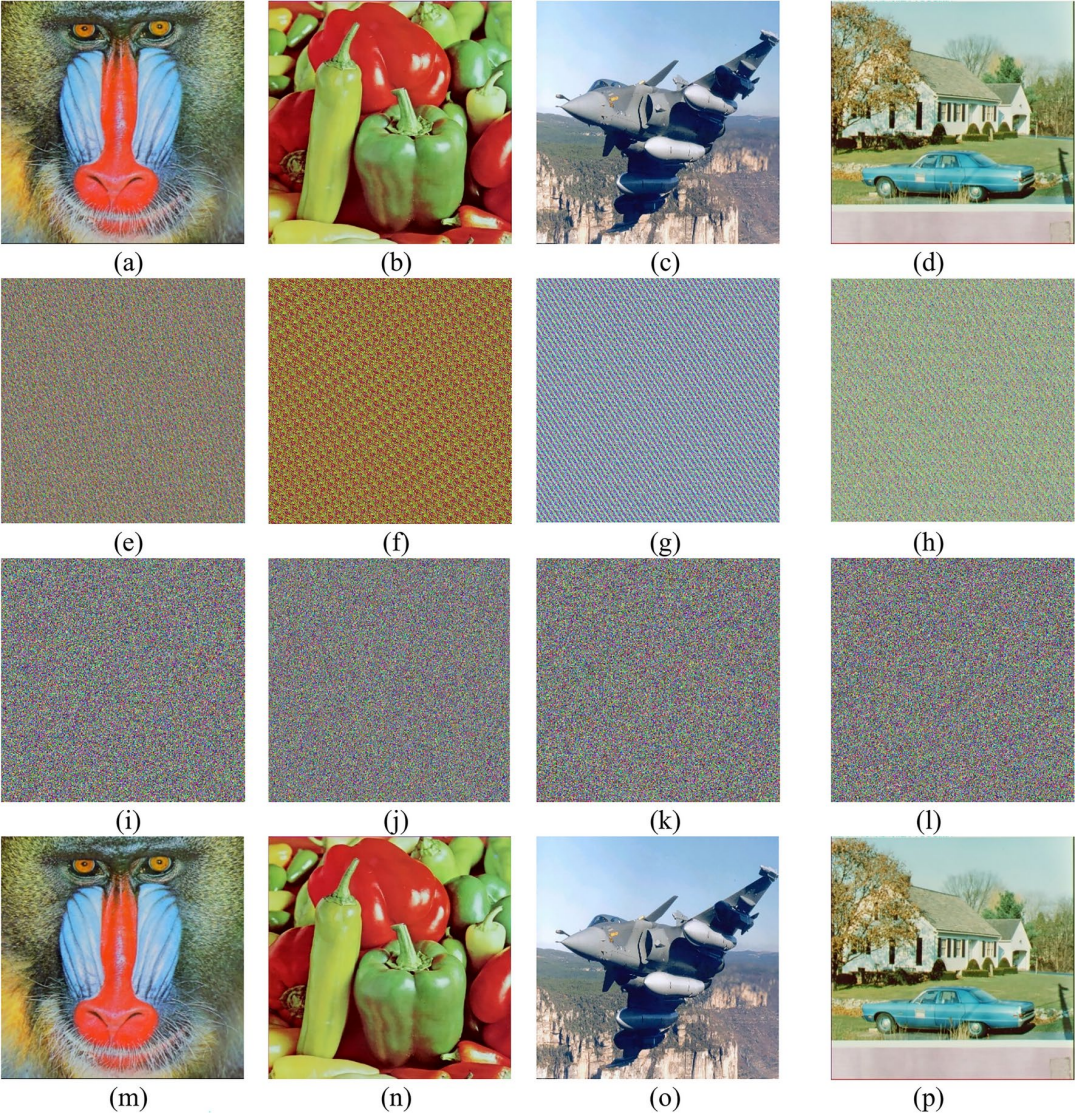
Plain images (**a**–**d**) of Baboon, Peppers, Rafal, and House shuffled images (**e**–**h**) using ACM. The encrypted images (**i**–**l**) using the proposed scheme and the deciphered images (**m**–**p**).

Multiple images were encrypted and decrypted to measure the efficiency of the proposed scheme. In this paper, a sample of standard 8-bit RGB images with size 256 × 256 × 3 for “Baboon” and “Peppers” and with size 512 × 512 × 3 for “Rafal” and “House” are encrypted and decrypted to assess the validation of the proposed scheme.The results presented in Fig. 2 prove that the ciphering algorithm effectively obscured the original images. The Fig. 2a–d show the original images, Fig. 2e–h show the shuffled images using Arnol Cat Map, and Fig. 2i–l shows the ciphered images while Fig. 2m–p shows the deciphered images.

The uniformly distributed histograms of the ciphered images and high entropy values close to 8, indicating strong randomness. Key sensitivity tests demonstrated that even minor variations in the encryption key resulted in substantial variations in the ciphered images, underscoring the algorithm’s robustness.

Furthermore, the algorithm exhibited excellent key space characteristics, with an adequately large key space making brute-force attacks computationally infeasible. The algorithm’s computational speed was also evaluated. The ciphering and deciphering processes were performed rapidly, highlighting the algorithm’s appropriateness for real-time applications. The efficiency of the algorithm, combined with its strong security features, making it a practical approach for secure image encryption in various scenarios.

The quality of the ciphered images was evaluated with two standard metrics: Peak Signal-to-Noise Ratio (PSNR) and Structural Similarity Index (SSIM). These evaluation metrics provide quantitative and perceptual evaluations of the similarity between the plain and ciphered images. PSNR is a widely used measure that quantifies the distortion introduced by encryption. It is defined based on the Mean Squared Error (MSE) among the plain image $I$ and the ciphered image $E$. The value of MSE is calculated as:

14 $$\text{MSE}=\frac{1}{M\times N}\sum_{i=1}^{M} \sum_{j=1}^{N} [I(i,j)-E(i,j){]}^{2}$$

where $M$, $N$ are the dimensions of the image, and $I(i,j)$ represents the pixel values of the plain image and $E(i,j)$ denotes the pixel values of ciphered images (Table 6). The PSNR is then derived from the MSE as follows:

15 $$\text{PSNR}=10\cdot {\text{log}}_{10}\left(\frac{{\text{MAX}}^{2}}{\text{MSE}}\right)$$

where MAX denotes the highest possible pixel value of the image (for instance, 255 in the case of an 8-bit image). A higher PSNR value signifies improved preservation of image quality, with values above 30 dB typically signifying acceptable levels of distortion. The SSIM comparing the two images, $x$ and $y$ is calculated as:

16 $$\text{SSIM}(x,y)=\frac{\left(2{\mu }_{x}{\mu }_{y}+{C}_{1}\right)\left(2{\sigma }_{xy}+{C}_{2}\right)}{\left({\mu }_{x}^{2}+{\mu }_{y}^{2}+{C}_{1}\right)\left({\sigma }_{z}^{2}+{\sigma }_{y}^{2}+{C}_{2}\right)}$$

where ${\mu }_{x}$ and ${\mu }_{y}$ denote the mean intensities, ${\sigma }_{x}^{2}$ and ${\sigma }_{y}^{2}$ represent the variances, and ${\sigma }_{xy}$ represents the covariance of the images $x$ and $y$. The constants ${C}_{1}$ and ${C}_{2}$ are used to stabilize the division, typically set based on the dynamic range of pixel values (Table 7). SSIM values take the range from − 1 to 1, where the perfect structural similarity is indicated by the value of 1, and values closer to zero indicate significant differences in image structure. Table 8 presents the SSIM and PSNR values, demonstrating the randomness of the encrypted images and the precision of the decrypted images in comparison to the plain images.

**Table 6:** Proposed ICC-PRNG framework mapping.

Parameters selection	Points generation	Points management	Random bit extraction	Notes
Selection of secure CC Use Gaussian number theory Exchange ${t}_{1} , {t}_{2}$	Increment index $P\left({t}_{1}\right)\oplus P\left({t}_{2}\right)=P\left({t}_{3}\right)$	Convert genrated points $P\left({t}_{n}\right)$ into binary form	Read the lower order 128 bits from $x, y$ coordinates Pad the extracted bits together	Various curves can be used *t*_*1*_* , t*_*2*_ is smaller than $N(p)$

**Table 7:** The NIST randomness tests of the binary output from the proposed ITM and ILM.

Tests	K_R_		K_G_		K_B_	
	P-V	P-P	P-V	P-P	P-V	P-P
Frequency	0.628	0.967	0.291	1	0.452	1
Block Frequency	0.381	1	0.175	1	0.363	1
Longest run of ones	0.532	1	0.727	1	0.582	1
Runs	0.642	1	0.542	0.985	0.735	1
Binary matrix rank test	0.4283	1	0.782	1	0.211	1
DFT	0.647	0.982	0.647	1	0.293	1
Non-overlapping template	0.389	0.986	0.332	0.983	0.211	0.985
Universal	0.294	1	0.029	0.921	0.234	0.978
Overlapping templates	0.897	0.989	0.028	0.986	0.135	0.958
Linear complexity	0.7419	1	0.7419	1	0.7392	1
Serial	0.8936	1	0.863	1	0.537	1
Random excursions variant	0.093	1	0.298	0.994	0.426	0.985
Approximate entropy	0.453	1	0.463	1	0.596	1
Cumulative sums (Forward)	0.537	1	0.293	1	0.508	1
Cumulative sums (Revere)	0.342	1	0.318	1	0.328	1
Random excursions	0.095	0.994	0.195	1	0.281	0.982
	Accepted		Accepted		Accepted

**Table 8:** Comparison between SSIM and PSNR of ciphered and deciphered images .

Image	Encrypted		Decrypted	
	SSIM	PSNR	SSIM	PSNR
Baboon	0.007984	8.7726	1	∞
Peppers	0.007803	8.3790	1	∞
Rafale	0.007517	8.0017	1	∞
House	0.007445	8.8882	1	∞

### NIST statistical test suite

The NIST SP-800-22 statistical test suite comprises 15 distinct tests designed to evaluate the randomness of the output bitstreams. If one of these tests does not pass, the bitstream is considered unsuitable for cryptographic use. The assessment results are interpreted using two main criteria: the Proportion of Passing sequences (P-P) and the Probability-Value distribution (P-V). For an ideal random sequence, the P-V must be close to 1, indicating randomness, whereas a P-V approaching 0 signifies non-randomness. The significance level, denoted by α, is typically set at 0.01. If the P-V meets or exceeds α, the sequence is considered random, passing the test with a confidence level of 99%. Conversely, if the P-V is below α, the sequence does not pass the test and is considered non-random.

**Fig. 3:**
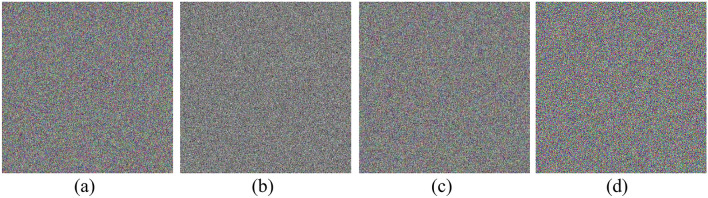
Proposed ICC-PRNG output RBS represented as color images.

NIST Test Results for ICC-PRNG: The designed ICC-PRNG was rigorously evaluated using the NIST evaluation test suite to assess the output randomness of system across the three color channels:$\text{ R}{\text{S}}_{\text{R}}$ for red, ${\text{RS}}_{\text{G}}$ for green, and ${\text{RS}}_{\text{B}}$ for blue. A total of 6,291,456 bits (equivalent to $24 \times {2}^{18}$) were generated for each sequence, corresponding to the bit count in a 512 × 512 color image. A single-bit alteration was made to the input to assess the sensitivity of the ICC-PRNG in generating a new bitstream. Despite this minor modification, the new bitstream still passed all 15 NIST randomness tests, demonstrating the PRNG’s robustness. The bitstreams were subsequently converted into different color images, as depicted in Fig. 3. Visual examination of these images corroborates the NIST results, revealing that the correlation between the bitstreams is approximately 0.00089, indicating a strong sensitivity to the one-bit alteration in the input. The results obtained from the test cases, confirming the consistent performance of the ICC-PRNG. Consequently, Table 7 presents the outcomes for representative test cases, illustrating the effectiveness of the proposed ICC-PRNG across different scenarios.

### Histogram analysis

**Fig. 4:**
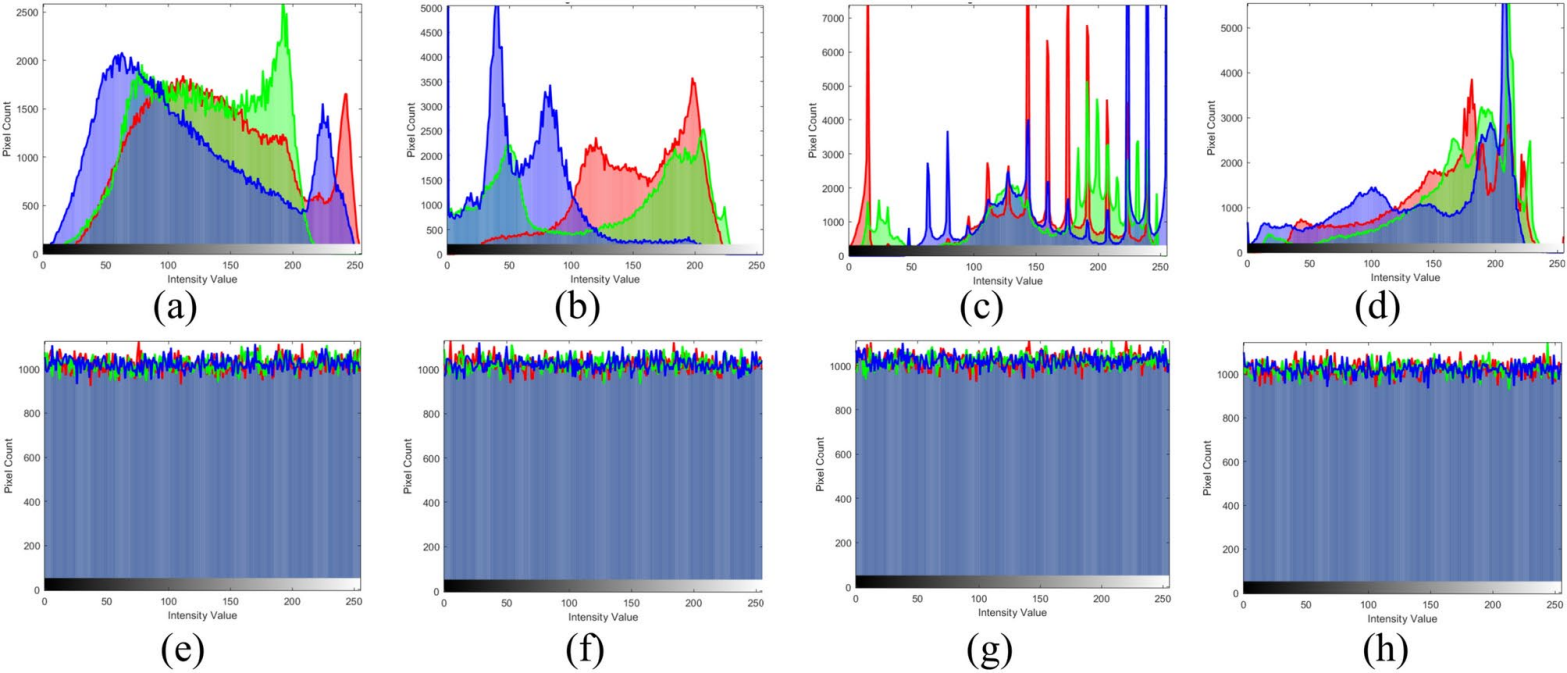
Histogram of plain color images in Fig. 2a–d and encrypted images in Fig. 2i–l.

A histogram provides a visual depiction of the frequency distribution of pixel intensity values within an image. For a secure encryption scheme, the histogram of the ciphered image must exhibit a uniform distribution, signifying that the pixel values are evenly spread across all possible intensity levels. This uniform distribution ensures that no visible patterns or statistical similarities exist between the plain and encrypted images, rendering it challenging for an adversary to get any significant information. By analyzing the differences between histograms of the original and encrypted images, one can visually and quantitatively assess the efficiency of the encryption process in eliminating recognizable features and achieving high security. Visual inspection of Fig. 4 reveals that the proposed scheme meets the goal of histogram analysis of: a. Baboon; b. Peppers; c. Rafal d. House color images; (a–d) are the histogram of plain images; (i–l) are the histogram of ciphered images; the results proves that it is no discernible patterns are present in the encrypted image. This is achieved through the integration of confusion and diffusion mechanisms, which enhances the proposed scheme robustness against to statistical attacks.

### Correlation coefficients of image pixels

The correlation coefficient measures the degree to which the pixel value: in the plain image are related to the pixel value in the ciphered image. For two sets of pixel values, $X$ (original image) and $Y$ (ciphered image), the correlation coefficient ($\rho$), is computed using this formula:

17a $$\rho =\frac{\text{cov}(X,Y)}{{\sigma }_{X}{\sigma }_{Y}}$$

Here, the covariance of $X$ and $Y$ is denoted by $\text{cov}(X,Y)$, while the standard deviations of $X$ and $Y$ is denoted by ${\sigma }_{X}$ and ${\sigma }_{Y}$, respectively. It is defined as:

17b $$\text{cov}(X,Y)=\frac{1}{N}\sum_{i=1}^{N} \left({X}_{i}-{\mu }_{X}\right)\left({Y}_{i}-{\mu }_{Y}\right)$$

where the number of pixel pairs is represented by $N$, ${X}_{i}$ and ${Y}_{i}$ are the intensity values corresponding pixels in the plain and cipherd images, ${\mu }_{X}$ and ${\mu }_{Y}$ are the mean values of $X$ and $Y$. The standard deviations are computed as follows:

17c $${\sigma }_{X}=\sqrt{\frac{1}{N}\sum_{i=1}^{N} {\left({X}_{i}-{\mu }_{X}\right)}^{2}},\,\, {\sigma }_{Y}=\sqrt{\frac{1}{N}\sum_{i=1}^{N} {\left({Y}_{i}-{\mu }_{Y}\right)}^{2}}$$

**Fig. 5:**
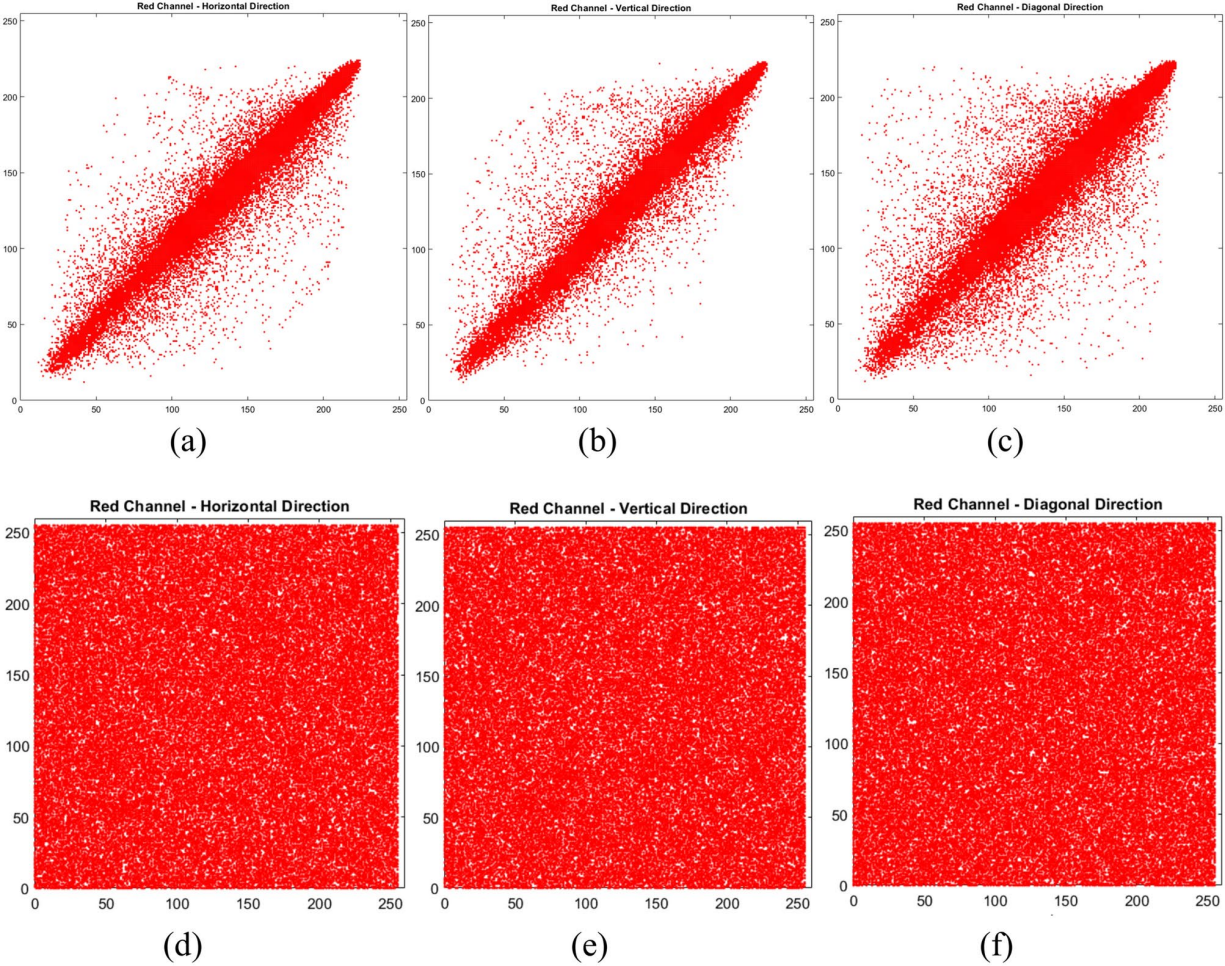
Pixel correlation for “Peppers” image adjacent pixels along Plain and Cipher Red channel.

The correlation coefficient $\rho$ varieties from − 1 to 1. A value of $\rho =-1$ signifies a perfect negative correlation, $\rho =1$ depicts a signifies positive correlation, and $\rho =0$ signifies no correlation. In a robust image encryption scheme, the correlation coefficient between the plain and ciphered images is anticipated to be near zero, demonstrating that the encryption has effectively obscured the pixel values and eliminated any discernible patterns. Figure 5 provides a visual representation of the correlation in the Horizontal (H), Vertical (V), and Diagonal (D) directions between the plain ‘Peppers’ Red channel image from Fig. 2b and its encrypted counterpart in Fig. 2j. However, for a robust encryption scheme, this correlation should be minimized, resulting in a correlation coefficient near to zero^55^. Table 9 compares the correlation coefficients of the proposed encryption scheme against other contemporary methods, demonstrating its ability to decorrelate pixel values and thereby enhance security. The correlation coefficients of plain images as illustrated in Table 9 are close to one while the encrypted images are near zero. The exhibited results prove the strong resistance against statistical-based attacks provided by the proposed method.

**Table 9:** Correlation coefficient of RGB tested images utilizes the proposed scheme.

Scheme	Image	Channel	H		N		D	
			Original	Cipher	Original	Cipher	Original	Cipher
Ours	Baboon (256 × 256)	R	0.9782	0.0005	0.9700	0.0008	0.9812	0.0003
		G	0.9538	− 0.0007	0.9737	− 0.0005	0.9866	− 0.0008
		B	0.9769	0.0023	0.9886	0.0017	0.8905	0.0001
	Peppers (512 × 512)	R	0.9103	0.0014	0.9212	0.0065	0.9342	0.0002
		G	0.8759	− 0.0011	0.7966	− 0.0005	0.8766	− 0.0035
		B	0.8972	0.0036	0.8725	0.0028	0.9724	0.0003
	Rafal (1024 × 1024)	R	0.9022	0.0015	0.9011	0.0005	0.9913	0.0065
		G	0.7897	− 0.0025	0.7957	− 0.0015	0.9917	− 0.0005
		B	0.8897	0.0078	0.8915	0.0008	0.8829	0.0027
Ref.^28^	House (512 × 512)	R	0.9506	− 0.0053	0.9562	− 0.0042	0.9193	− 0.0081
		G	0.9384	− 0.0028	0.9456	− 0.0076	0.8863	0.0021
		B	0.9721	0.0050	0.9717	− 0.0014	0.9482	− 0.0026
Ref.^50^	Baboon (512 × 512)	R	0.9199	0.0025	0.8637	0.0003	0.8489	− 0.0008
		G	0.8596	− 0.0011	0.7588	− 0.0005	0.7265	0.0019
		B	0.9041	− 0.0043	0.8775	0.0014	0.8346	0.0007
	Lena (256 × 256)	R	0.9473	0.0035	0.9754	− 0.9672	0.9229	− 0.0058
		G	0.9332	− 0.0073	0.0039	− 0.004	0.9084	0.00008
		B	0.9142	0.0035	0.9524	− 0.0039	0.887	0.0058

### Differential attack analysis

This type of attack assesses how minor changes in the plain input image impact the ciphered output. Two common metrics used in this context are the Number of Pixels Change Rate (NPCR) and the Unified Average Changing Intensity (UACI). The NPCR Computes the proportion of pixels that differ between two ciphered images derived from slightly different original images and is given by:

18a $$\text{NPCR}=\frac{\sum_{i=1}^{M} \sum_{j=1}^{N} D(i,j)}{M\times N}\times 100\text{\%}$$

where the dimensions of the image are represented by $M$ and $N$, and $D(i,j)$ is a binary indicator function that equals 1 if the pixel values at the location $(i,j)$ in the two ciphered images are different, and 0 otherwise. The UACI computes the average differences in pixel intensity between the encrypted images and is defined by the following formula:

18b $$\text{UACI}=\frac{1}{M\times N}\sum_{i=1}^{M} \sum_{j=1}^{N} \frac{\left|{C}_{1}(i,j)-{C}_{2}(i,j)\right|}{255}\times 100\text{\%}$$

where the pixel values of the two ciphered images at position $(i,j)$ are denoted by ${C}_{1}(i,j)$ and ${C}_{2}(i,j)$. For a robust image encryption scheme, the theoretical values of NPCR must be close to $99.6094\text{\%}$, and UACI should be approximately $33.4635\text{\%}$. High values of NPCR and UACI indicate that the encryption algorithm is very sensitive to small variations in the input, thus providing strong security against differential attacks. To assess the NPCR and UACI performance of the proposed algorithm, a single pixel in the plain image was arbitrarily selected and its value was modified. The NPCR and UACI were then calculated using the ciphered image derived from the original and modified images. The outcomes of these calculations for the tested images are outlined in Table 10, accompanied by a comparative assessment of other recent schemes.

**Table 10:** NPCR and UACI values evaluation and comparison.

RGB image	RGB image	Component	NPCR (%)	UACI (%)
Peppers (256 × 256)	Ours	R	99.63	33.51
		G	99.61	33.56
		B	99.63	33.47
	Ref. ^43^	R	99.60	33.40
		G	99.60	33.45
		B	99.61	33.36
Baboon (512 × 512)	Ref.^49^	R	99.76	33.34
		G	98.96	33.24
		B	99.96	33.31
	Ref. ^50^	R	99.63	33.49
		G	99.63	33.40
		B	99.64	33.45
	Ours	R	99.62	33.47
		G	99.61	33.42
		B	99.64	33.44

### Key space

Refers to the entire set of possible keys that can be used within the encryption system. To safeguard against brute-force and naive attacks, the key space must be large enough. Evaluating the key space involves examining both key sensitivity and the overall number of potential keys. To ensure robust security against such attacks, the encryption algorithm should have a minimum key space of at least ${2}^{128}$. The key space of the proposed encryption scheme is sufficiently large to withstand the naive attack, and it can be calculated as 2^128^ × 2^128^ × 2^128^ 2^256^ × 2^48^ = 2^688^, where the first and second 128 bits are given from the ${t}_{1}, {t}_{2}$ initial parameters, the third 128 bits is from the private number $d$, the fourth 256 bits is from the prime $p$, $q$ and the last 48 bits are from control parameter the Arnold Cat Map. The implementation of the GCC enlarge the key space, and this is achieved with simple point mathematical operations. Table 11 compares the proposed system key space with various existing schemes.

**Table 11:** Comparison of key space.

Ours	Ref.^42^	Ref.^6^	Ref.^40^	Ref.^20^	Ref.^28^	Ref.^50^
Key space size 2^688^	2^194^	2^262^	2^328^	2^398^	2^448^	2^504^

### Key sensitivity

**Fig. 6:**
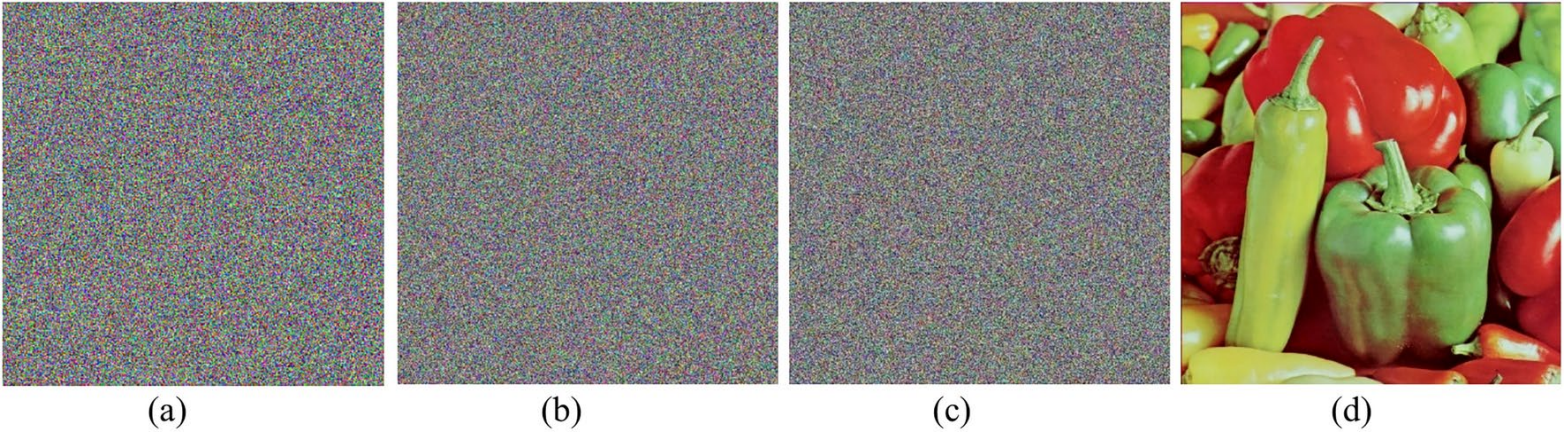
Key Sensitivity: a Decryption with one bit alter in (**a**) plain image. (**b**) conic key. (**c**) base point G. (**d**) Correct Key decryption.

A robust encryption system must demonstrate a strong key sensitivity, meaning that even a slight alteration in the system key should produce a significantly different encrypted image. This property ensures that unauthorized users cannot decrypt the image without the exact key, thereby enhancing security. Key sensitivity is typically evaluated by ciphering the identical image with slightly altered keys and comparing the encrypted images. Effective key sensitivity is demonstrated when the encrypted images show no correlation, indicating that the encryption process is highly dependent on the precise key used. Figure 6 demonstrates the impact of different key variations on the recovered image. In Fig. 6a,b, the image is recovered after altering the initial values of the ICC-PRNG. Figure 6c shows the recovered image when a single-bit in both the original image and the initial key is modified. For reference, the properly deciphered image is also displayed in Fig. 6. The results demonstrate the strong key sensitivity of the proposed algorithm.

### Computational speed

Evaluates the time needed to execute the encryption and decryption of an image, influencing the algorithm’s practicality for real-time applications. Faster computational speed indicates higher efficiency, making the algorithm more suitable for scenarios requiring rapid processing, such as live video streaming or large-scale image datasets. The computational speed is typically assessed by benchmarking the algorithm on standard hardware and measuring the time taken to process images of varying sizes. An efficient image encryption algorithm should balance high security with minimal computational overhead, ensuring that it can be deployed effectively in performance-sensitive environments. Table 12 presents a comparative evaluation of execution time for various image encryption schemes across different dimensions. The proposed scheme achieves significantly lower encryption time (0.025 s for 256 × 256 × 1 and 0.152 s for 512 × 512 × 1) while integrating multi-hard problem security (CC-DLP, GCC-IFP, and CC-IFP) and digital signature functionality. Compared to Ref.^5^, the proposed scheme demonstrates a 97.26% time saving, while Ref.^43^ exhibits notably higher computational overhead. Although Ref.^35^ offers marginally faster encryption for larger images, it lacks public-key infrastructure and digital signatures, limiting its application scope. The critical evaluation indicates that the proposed scheme provides a balanced trade-off between computational efficiency and robust multi-layered security, thereby justifying its applicability in real-time and resource-constrained environments. The encryption time is a sum of the all system execution time for initial parameter exchange, ICC-PRNG output, image shuffling and Xor operation.

**Table 12:** Execution time and hardness comparison.

Ref	Image dimensions	Encryption time (s)	Time saving comparison (%)	Hard problems utilized	Functionality
Our	256 × 256 × 1	0.025		CC-DLP, GCC-IFP, CC-IFP	Encryption + Complex digital signature
Our	512 × 512 × 1	0.152			
Ref.^5^	256 × 256 × 1	0.9120	97.26	Not utilized	Encryption only
Ref.^35^	512 × 512 × 1	1.42	− 7.04	Not utilized	Encryption only
Ref.^40^	512 × 512 × 1	1.1091	86.29	EC-DLP, EC-IFP	Encryption + Digital signature
Ref.^43^	512 × 512 × 1	3.78	95.97	EC-DLP	Encryption only

### Entropy analysis

Entropy evaluates the unpredictability within the pixel distribution of an image. A higher entropy value signifies a more secure and less predictable ciphered image. The entropy $H(X)$ of an image $X$ is expressed by:

19 $$H(X)=-\sum_{i=1}^{n} p\left({x}_{i}\right){\text{log}}_{2}p\left({x}_{i}\right)$$

where the likelihood of occurrence of each pixel intensity value ${x}_{i}$ in the image is represented by $p\left({x}_{i}\right)$. For an 8 -bit grayscale image, the highest entropy is 8 bits, indicating a perfectly random image. The more closely the entropy of the encrypted image approaches this maximum value, the more secure the encryption is considered to be. Table 13 shows that the results of the proposed method are consistently close to 8, outperforming the results of the existing method outlined in Table 14. This highlights the proposed method’s strong resistance to statistical analysis.

**Table 13:** Entropy of original and cipher image.

Image	Component	Original entropy	Cipher entropy
Baboon	R	7.7261	7.9994
	G	7.5724	7.9992
	B	7.7426	7.9992
Peppers	R	7.5081	7.9994
	G	7.4298	7.9993
	B	6.6941	7.9992
Rafal	R	6.7388	7.9992
	G	6.7898	7.9991
	B	6.3127	7.9993
House	R	7.2471	7.9993
	G	7.4890	7.9992
	B	6.9734	7.9993

**Table 14:** Comparison of Entropy with recent schemes.

Scheme image	Entropy	
	Baboon	Peppers
Ours	7.9993	7.9997
Ref^6^	7.9993	7.9993
Ref^20^	7.9991	7.9991
Ref^28^	7.9993	7.9994
Ref^42^	7.9993	7.9993
Ref^43^	–	7.9970
Ref^49^	7.9952	–

### Avalanche effect

In a robust image encryption system, the avalanche effect can be quantified by the percentage of differing binary bits between two ciphertexts ${C}_{1}$ and ${C}_{2}$, generated from slightly different plaintexts ${P}_{1}$ and ${P}_{2}$. Ideally, this difference should be around 50%, which is mathematically expressed as:

20 $$\text{Avalanche effect }=\left(\frac{1}{n}\sum_{i=1}^{n}\Delta \left({C}_{1}[i],{C}_{2}[i]\right)\right)\times 100\text{\%}$$

where $\Delta \left({C}_{1}[i],{C}_{2}[i]\right)$ represents the bitwise difference between corresponding bits of the ciphertexts, and $n$ is the total number of bits. A result close to $50\text{\%}$ indicates a strong avalanche effect, signifying that the ciphering process is highly sensitive to minor input alteration, thereby enhancing security. Table 15 presents the result of the avalanche effect while changing just a one-bit in the initial key and original image. The avalanche effect of the proposed scheme presents good results.

**Table 15:** Avalanche effect.

Image	Single-bit alteration in initial key (%)	Single-bit alteration in plain image (%)
Baboon	50.0423	49.9896
Peppers	49.9867	50.1024
Rafal	49.8324	50.0239
House	49.9327	49.8795

### Choosen plain text attack

**Fig. 7:**
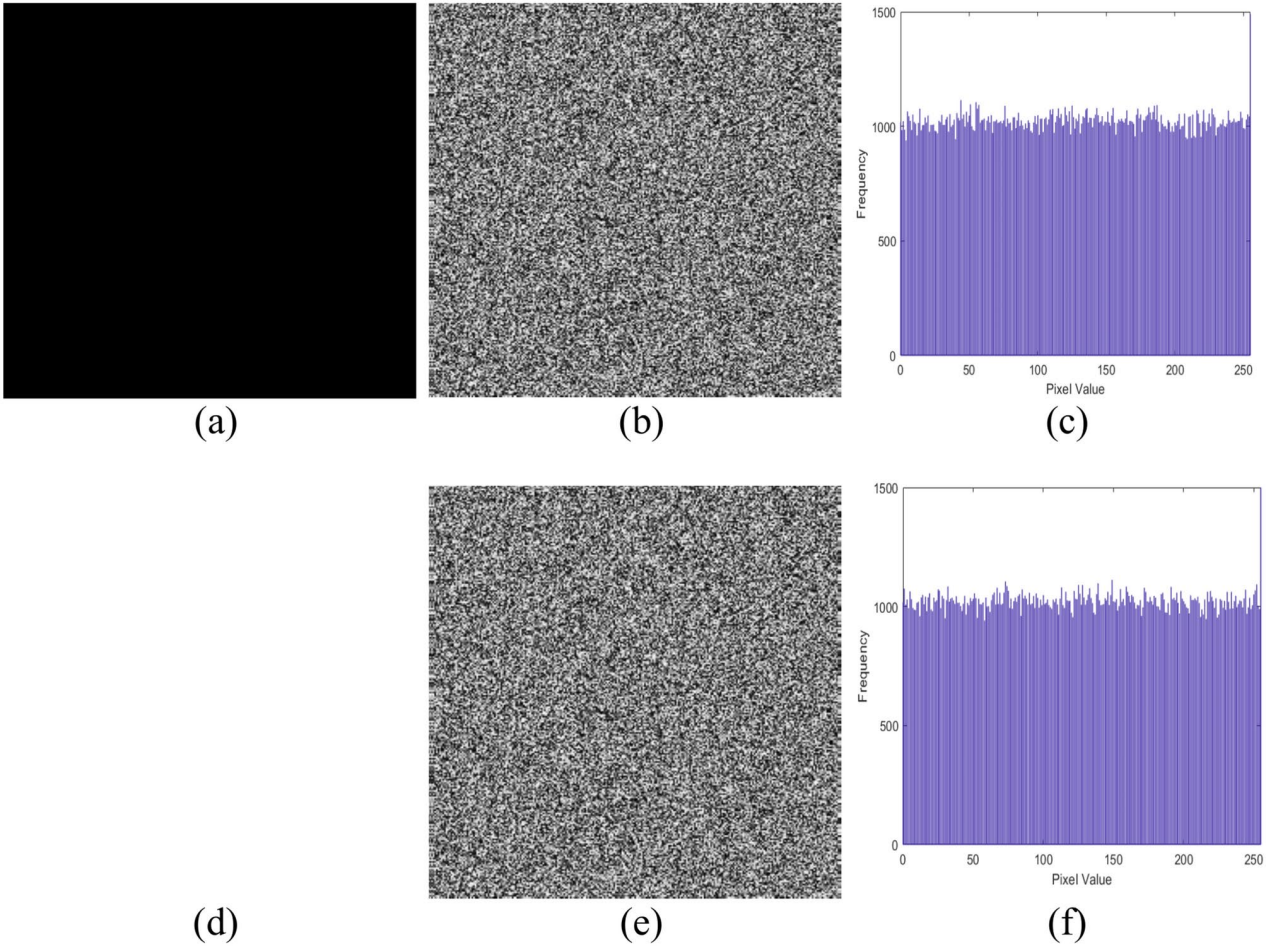
All black image (**a**) and its encrypted image, histogram (**b**–**c**); white image (**d**) and its encrypted image, histogram (**e**–**f**).

In a chosen-plaintext attack, an attacker can obtain both plaintext and ciphertext pairs. Specifically, if the attacker uses a black image (where all pixels are zero) as the chosen plaintext, they can determine the RBS used in encryption, since XORing the RBS with the black image yields the RBS itself. To mitigate this vulnerability, the proposed algorithm uses the hash of the plain image as the initial key for the ICC-PRNG. This approach guarantees that even the smallest modification in the image produces a different hash value, leading to a completely distinct sequence of output bits from the ICC-PRNG. As a result, the RBS generated for separately color channel (R, G, B) are unique to every image, significantly enhancing the system security. Moreover, an adversary in possession of one or more plain-cipher image pairs would be unable to produce the RBS for a new encrypted image, as the hash-based key is inherently image-specific. Additionally, even with knowledge of the RBS, the private key $d$ cannot be derived, further safeguarding the communication. Figure 7 shows the black and white images followed by its encrypted images and histogram.

## Noise attack

### Gaussian noise

**Fig. 8:**
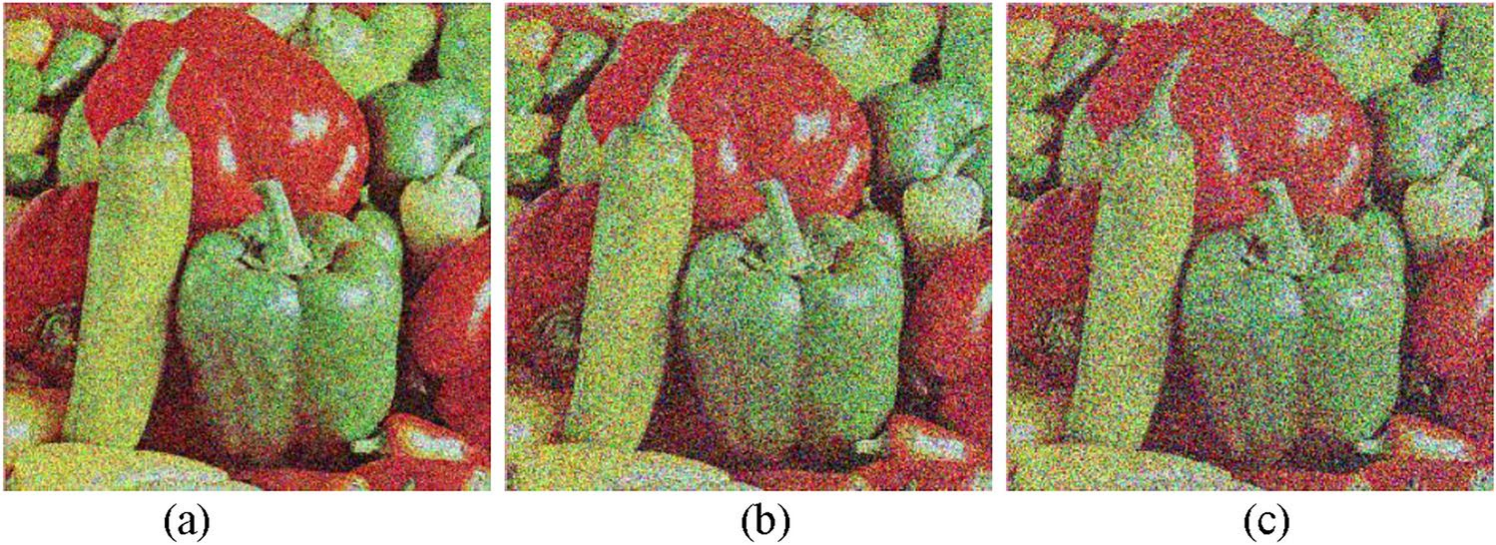
Peppers decrypted image (**a**–**c**) after encrypted image exposure to 0.1, 0.2 and 0.3 of Gaussian noise.

Gaussian noise simulates the impact of random, additive disturbances that can occur during image transmission or storage. To assess the strength of the encryption algorithm, Gaussian noise with varying mean and variance values was added to the ciphered image, followed by the decryption process. Different intensities of Gaussian noise (0.1, 0.2, 0.3, and 0.4) were applied to the encrypted image before decryption. The results, illustrated in Fig. 8, demonstrate that the proposed encryption scheme can overcome Gaussian noise, preserving a recognizable level of image quality. Although the noise introduces subtle distortions, the deciphered images maintain the essential features of the original, underscoring the effectiveness of the used method in maintaining data integrity under adverse conditions.

### Salt and pepper noise attack

**Fig. 9:**
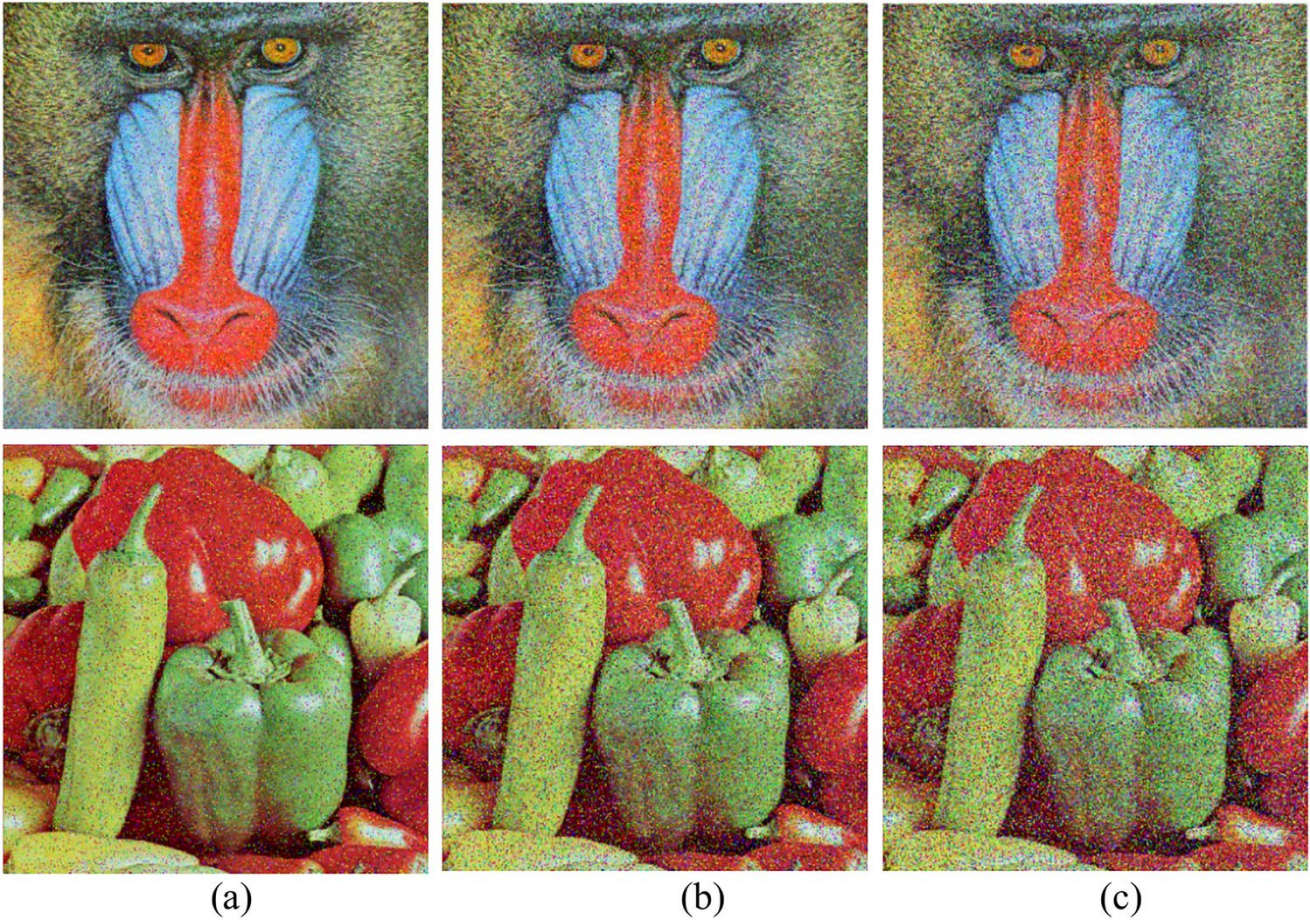
Baboon and peppers decrypted image after adding (**a**) 10%, (**b**) 20% and (**c**) 30% salt-pepper noise to encrypted image.

The salt-and-pepper noise attack, characterized by the introduction of random black (pepper) and white (salt) pixels, simulates data corruption or tampering by embedding extreme values (0 and 255) into an encrypted image. This type of noise, applied post-encryption, assesses the encryption algorithm’s resilience and its ability to recover the original image despite substantial disturbances. The effects of varying noise levels (10%, 20%, and 30%) on the ciphered images are illustrated in Fig. 9a–c. Nevertheless, the proposed encryption scheme demonstrates notable robustness, maintaining a perceptually recognizable decrypted image even under high noise conditions. This effectiveness highlights the scheme’s ability to preserve image integrity and ensure reliability and security despite significant noise-induced disruptions.

### Occlusion attack

**Fig. 10:**
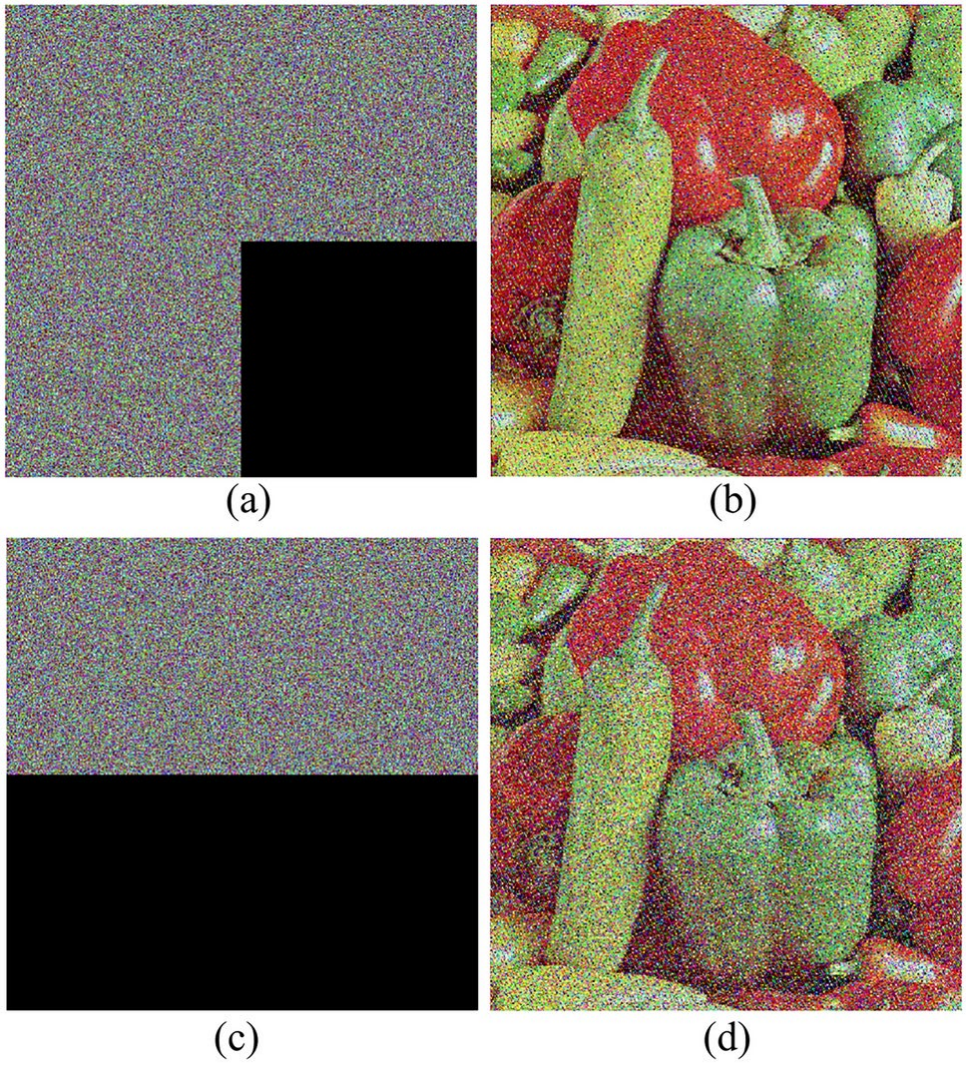
Decrypted image (**b**–**d**) after 25%, 50% occlusion attack of encrypted image in (**a**–**c**), respectively.

An occlusion attack deliberately blocks a portion of the ciphered image to simulate data corruption, testing the strength of the encryption scheme in reconstructing the original image despite missing data. In the proposed scheme, occlusion attacks were simulated by replacing 25% and 50% of the ciphered image with black pixels. The subsequent decryption process demonstrated the encryption scheme’s ability to recover from such disruptions. Figure 10 presents the retrieved image after exposure to the occlusion attack, where visual inspection indicates that the occluded region has spread across the encrypted image due to the deshuffling effect of the Arnold cat map. The outcomes confirm that the proposed encryption approach maintains a recognizable level of image quality even under significant data loss, affirming its robustness and reliability in adverse conditions.

### Rotation attack

**Fig. 11:**
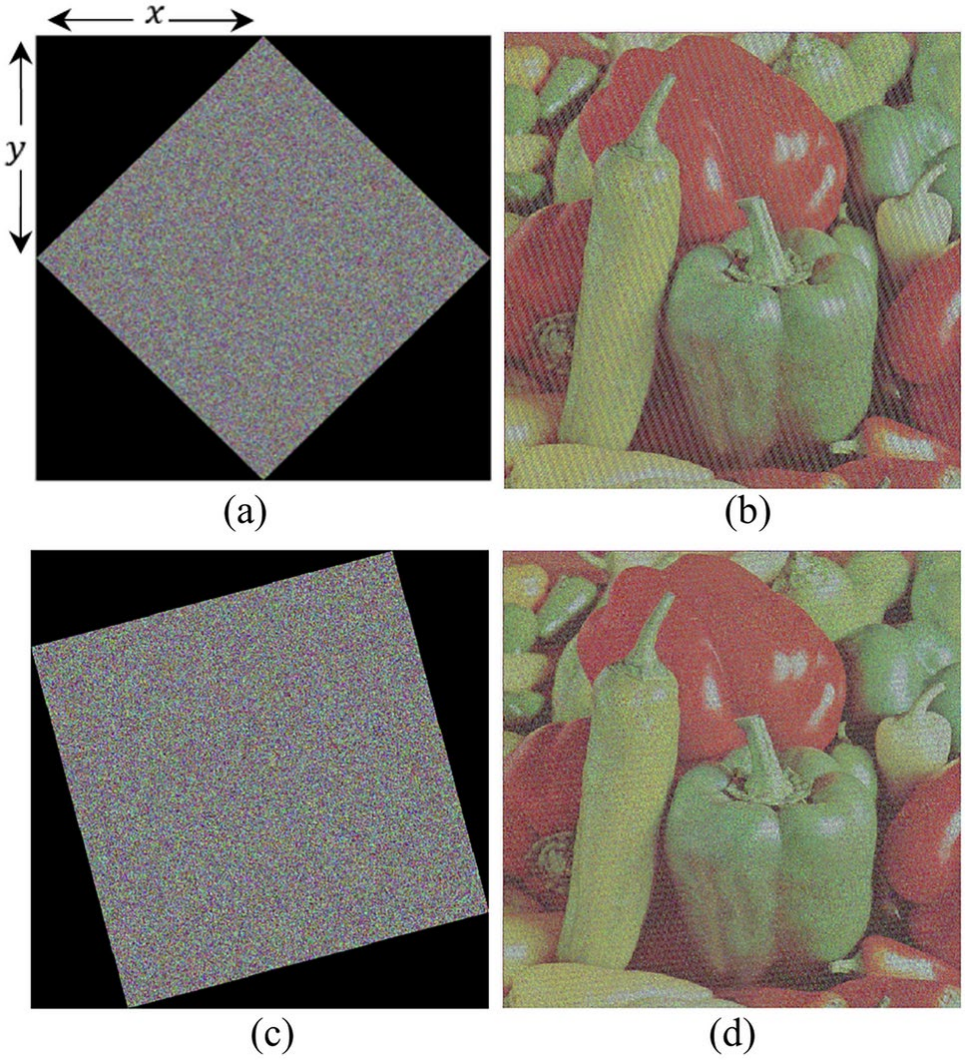
Decrypted image (**b**–**d**) after ${15}^{\circ }$, ${45}^{\circ }$ rotation attack of encrypted image in (**a**–**c**), respectively.

A rotation attack evaluates the encryption scheme’s robustness against geometric transformations by rotating the encrypted image at specific angles. In the proposed scheme, rotation attacks of ${15}^{\circ }$ as shown in Fig. 11a and ${45}^{\circ }$ as shown in Fig. 11c were applied to the encrypted image. After these rotations, the image was decrypted, and the rotation was reversed to restore its original orientation. The practical reversal process involved locating the first non-zero pixel value along the vertical and horizontal directions. For the vertical direction, the distance from the top of the image was measured and represented by ($y$), while for the horizontal direction, the distance from the rightmost edge to this pixel was measured and represented by ($x$) as shown in Fig. 11a. This method ensured accurate restoration of the image’s original orientation after decryption. To reverse the rotation, the angle of rotation was calculated using the arctangent formula $\theta = arctan(x/y)$, where $x$ and $y$ are the measured dimensions of the rotated image. Based on the computed angle, the reverse rotation was adjusted as follows:

- If ${0}^{\circ }<\theta <{90}^{\circ }$, the reverse angle $RR$ is $-\theta$.
- If ${90}^{\circ }<\theta <{180}^{\circ }$, the reverse angle is $-\left({180}^{\circ }-\theta \right)$.
- If ${180}^{\circ }<\theta <{270}^{\circ }$, the reverse angle is $-\left({270}^{\circ }-\theta \right)$.
- If ${270}^{\circ }<\theta <{360}^{\circ }$, the reverse angle is $-\left({360}^{\circ }-\theta \right)$.

The results, revealed in Fig. 11b-d, show that the used method is robust against rotation attacks, maintaining the essential features of the plain image even after rotation and decryption. This resilience underscores the encryption scheme’s effectiveness in preserving data integrity and security under geometric transformations.

## Complexity evaluation and security outcomes

Operations involving complex numbers offer enhanced computational efficiency due to the independent processing of real and imaginary components. For example, when multiplying two complex numbers, $(a+bi)(c+di)$, the real and imaginary parts are computed as $ac-bd$ and $ad+bc$, respectively. This separation simplifies implementation and allows hardware to process smaller values relative to equivalent operations on rational integers. Specifically, the magnitude of operands in complex arithmetic is approximately half that of rational arithmetic, resulting in reduced processing time and improved throughput.

From a computational complexity perspective, conventional long multiplication for rational integers has a complexity of $O\left({n}^{2}\right)$, whereas the corresponding operation on Gaussian integers exhibits a complexity of $O\left((n/2{)}^{2}\right)=O\left({n}^{2}/4\right)$, thereby reducing computational cost by $75\text{\%}$. Even with parallel multipliers calculating the real and imaginary components concurrently, the total complexity remains $O\left({n}^{2}/4\right)$, indicating a significant efficiency gain. Moreover, advanced multiplication techniques can further minimize the computational overhead. These advantages allow cryptographic systems to be implemented on less expensive, lower-complexity hardware while achieving substantial speed improvements, up to fourfold increases without compromising accuracy or security.

### Numerical example

An example using small parameters to proof the validity of the proposed complex authentication and complex digital signature scheme.

#### Complex parameter selection and authentication

The first step between the sender and receiver is the conic curve choice and parameter selection where $a=2$ and $b=1$. Bothe sender A and receiver B apply the complex key sharing steps presented in Sect. "Conic curve cryptography" and complex Digital signature as follows:

$$\begin{aligned} & p = 7 + 8i,q = 6 + i,n = p \times q = 34 + 55i \hfill \\ & N\left( p \right) = 113,\quad N\left( q \right) = 37,\quad N\left( n \right) = 4181 \hfill \\ &\left( {N\left( p \right) - 1} \right)\left( {N\left( q \right) - 1} \right) = 4032,\quad e = 1123,\quad d = 1867 \hfill \\ & r = 57,s = 19,N_{n} = 2 \times 57 \times 19 = 2166 \hfill \\ & t_{1} = 2965\quad T = \left( {2117,1224} \right),\quad t_{2} = 2649\quad G = \left( {1516,2124} \right) \hfill \\ & {\text{Convert }}G{\text{ and }}T{\text{ to complex form }} \to T^{\prime } = \left( {\left( {21 + 17i} \right),\left( {12 + 24i} \right)} \right),G^{\prime } = \left( {\left( {15 + 16i} \right),\left( {21 + 24i} \right)} \right) \hfill \\ & {\text{Get encrypted complex }}T^{\prime } {\text{ and }}G^{\prime } \to T^{{\prime \prime }} = \left( {\left( { - 10 + 41i} \right),\left( { - 35 - 14i} \right)} \right),G^{{\prime \prime }} = \left( {\left( {25 - 21i} \right),\left( { - 15 - 1i} \right)} \right) \hfill \\ & {\text{Decrypt complex }}T^{\prime \prime} {\text{ and }}G^{\prime \prime} \to T^{\prime} = ((21 + 17i),\left( {12 + 24i} \right)),G^{\prime } = (\left( {15 + 16i} \right),\left( {21 + 24i} \right)) \hfill \\ & {\text{Concatenate the modulas of }}T^{\prime} ,G^{\prime} {\text{ to retrive }}T,G \to T = \left( {2117,1224} \right),G = \left( {1516,2124} \right) \hfill \\& {\text{Retrive}}t_{1} = 2965fromT = \left( {2117,1224} \right)and,\;\;t_{2} = 2649from\,\;G = (1516,2124) \hfill \\ \end{aligned}$$

#### Complex digital signature generation 

The parameters of the complex digital signature is selected then the steps in Sect. "Encryption and complex digital signature generation" from 4–5 is applied

$$\begin{aligned} & p = 4 + i,q = 2 + 3i,n = p \times q = 5 + 14i \\ & N\left( p \right) = 17,N\left( q \right) = 13,N\left( n \right) = 221 \\ & \left( {N\left( p \right) - 1} \right)\left( {N\left( q \right) - 1} \right) = 192,e = 5,d = 77 \\ & r = 9,s = 7,N_{n} = 2 \times 9 \times 7 = 126 \\ & Z = 10 \\ & V = \alpha + \beta \\ & V = 5,\alpha = 2\beta = 3 \\ & Z^{V} mod\,N\left( n \right) = 10^{5} mod\;221 = 108 \\ & {\text{Z}}^{\beta } mod\,N\left( n \right) = 10^{3} mod\,\,221 = 116 \\ & G = \left( {12,87} \right) \\ & {\text{ Compute }}U_{A} {\text{as in Eq}}.8 \to U_{A} = 108.\left( {12,87} \right)mod\,\,221 = \left( {14,53} \right) \\ & {\text{ Convert }}U_{A} {\text{ to complex form }} \to U_{A}^{\prime} {\text{then enrypt it using }}e{\text{ to get }}U_A^{\prime \prime} \to U_A^{ \prime } = \left( {\left( {1 + 4i} \right),\left( {5 + 3i} \right)} \right),U_{A}^{{\prime \prime }} = \left( {\left( { - 2} \right),\left( { - 6 - 3i} \right)} \right) \\ & {\text{ Compute }}R{\text{as in Eq}}.9{\text{a }} \to R = 116.\left( {12,87} \right)mod\,221 = \left( {63,32} \right) \\ & {\text{ Convert }}R{\text{ to complex form }} \to R^{\prime} {\text{then enrypt it using }}e{\text{ to get }}R^{{\prime \prime }} \to R^{\prime} = \left( {\left( {6 + 3i} \right),\left( {3 + 2i} \right)} \right),R^{{\prime \prime }} = \left( {\left( { - 4 + 5i} \right),\left( {7 + i} \right)} \right) \\ & {\text{Assume }}Hash\left( C \right) = 15. \\ & {\text{ Compute }}S = \left( {\alpha + Hash\left( C \right) + N\left( n \right)} \right)mod\,N_{n} = \left( {2 + 15 + 221} \right)\;mod\;126 = 112 \\ & {\text{ The Sender A send the Public key }}U_{A}^{\prime\prime} {\text{ in complex form and the complex signature }}(S,R^{\prime\prime}) \\ \end{aligned}$$

#### Complex digital signature verification

The receiver B receive the ciphered image and take the Hash of the received image then do the following:

$$\begin{aligned} & Hash\left( C \right) = 15. \\ & {\text{Recive the complex encrypted public key}}\;U_{A}^{{\prime \prime }} = \left( {\left( { - 2} \right),\left( { - 6 - 3i} \right)} \right){\text{and the complex digital signature}} \\ & \left( {S,R^{{\prime \prime }} } \right) = \left( {112,\left( {\left( { - 4 + 5i} \right),\left( {7 + i} \right)} \right)} \right) \\ & {\text{Decrypt the complex numbers to retrive }}U_{A} ,R \\ & {\text{Decrypt complex}}\,U_{A}^{{\prime \prime }} {\text{ and }}R^{{\prime \prime }} \to U_{A}^{\prime } = \left( {\left( {1 + 4i} \right),\left( {5 + 3i} \right)} \right),\;R^{\prime } = \left( {\left( {6 + 3i} \right),\left( {3 + 2i} \right)} \right) \\ & {\text{Concatenate the modulas of }}U_{A}^{\prime } ,\;R^{\prime } {\text{ to retrive }}U_{A} ,\;R \to U_{A} = \left( {14,53} \right),R = \left( {63,32} \right) \\ & {\text{Compute }}D_{1} = 10^{{112}} \left( {63,32} \right)mod\;221 = \left( {{\text{76,33}}} \right),D_{2} = 10^{{15 + 221}} \left( {14,53} \right)\;mod\;221 = \left( {{\text{76,33}}} \right) \\ & {\text{If }}D_{1} = D_{2} \,{\text{the receiver B confirm the encrypted image and reverse the process of encryption}} \\ & {\text{If }}D_{2} \ne D_{1} \,{\text{the receiver B reject the encrypted image and end the process}} \\ \end{aligned}$$

### Security outcomes

The proposed scheme leverages CCC, which demonstrates superior security performance compared to traditional approaches. The algorithm is designed to mitigate several well-known vulnerabilities associated with ECC, including Anomalous Curves Attacks, Supersingular Curves Attacks, and general attacks^46^. By relying on three distinct and computationally difficult mathematical problems, the scheme forces attackers to engage in a series of complex analyses rooted in both rational and Gaussian number theory. A key feature of this system is its dynamic key exchange mechanism, where in the encryption process is continuously adapted based on the hash of the plaintext image. This dynamic nature, supported by the properties of the conic curve, ensures that even minimal deviations in the correct key result in substantial alterations to the entire encryption sequence. The simplicity of the addition operation on the conic curve, combined with the repeated use of this operation to perform multiplication, makes the process more efficient than traditional ECC methods. Furthermore, the point addition and multiplication operations required to compute the signature (S, R) are computationally efficient, with some operations capable of being precomputed offline. The integration of rational and Gaussian number theory enhances the robustness of the algorithm, rendering it particularly suitable for highly sensitive communications, where secure image transmission is paramount.

## Conclusion

This paper presents a robust, efficient, and secure image encryption and authentication scheme leveraging the strengths of CCC combined with complex number theory. By integrating multi-hard cryptographic problems, the proposed approach achieves enhanced security, computational efficiency, and resilience against various attacks, making it well-suited for critical applications such as military communications and healthcare.

The key innovations of this work include the development of an Iterative conic curve pseudorandom number generator (ICC-PRNG), which passes rigorous statistical randomness tests, and a hybrid digital signature mechanism addressing authentication, integrity, and non-repudiation requirements. Additionally, the combination of XOR-based encryption with Arnold Cat Map-based permutation ensures high confidentiality while maintaining computational efficiency.

The proposed scheme not only addresses inherent vulnerabilities in traditional cryptographic methods, such as side-channel and ECC-specific attacks, but also surpasses existing techniques in both security and practical performance. Comparative analysis demonstrates its ability to provide long-term security by leveraging the computational advantages of CCC over Gaussian integers.

Future work may explore optimizing the scheme further for deployment in resource-constrained environments, such as IoT devices, and extending its applicability to other multimedia formats. The proposed approach establishes a promising foundation for advancing cryptographic techniques to meet the evolving demands of secure and efficient digital communication.

## Data Availability

Similar to other studies in this research direction, we utilized several standard images, including Peppers, House, Baboon, and Rafal, among others. These images were obtained from the USC-SIPI image database. The datasets are publicly available and widely used for benchmarking image processing and encryption algorithms^70^.
